# Micellar Catalysis as a Tool for C–H Bond Functionalization toward C–C Bond Formation

**DOI:** 10.1021/acs.organomet.2c00309

**Published:** 2022-08-15

**Authors:** Elena Borrego, Ana Caballero, Pedro J. Pérez

**Affiliations:** Laboratorio de Catálisis Homogénea, Unidad Asociada al CSIC, CIQSO Centro de Investigación en Química Sostenible and Departamento de Química, Universidad de Huelva, Campus de El Carmen, 21007 Huelva, Spain

## Abstract

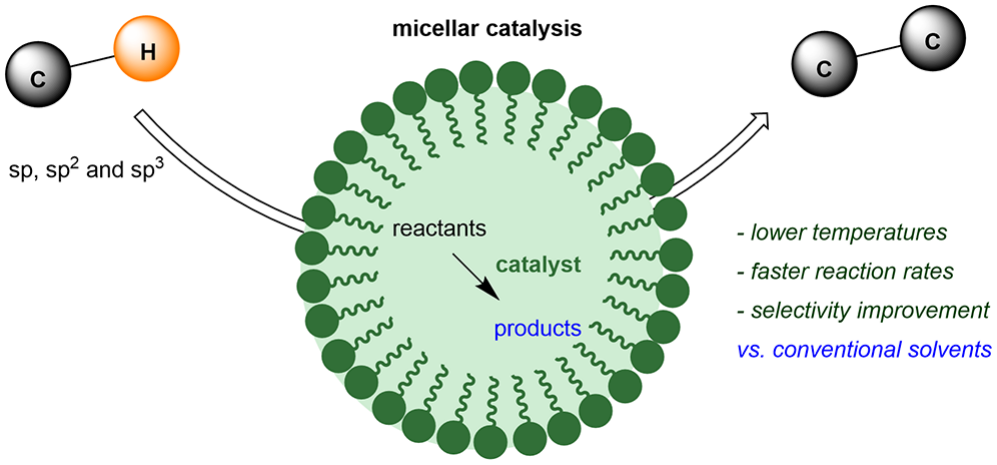

Micelles generated upon dissolving surfactants in water can be employed as nanovessels for catalytic transformations, in the so-called micellar catalysis methodology. This review is focused on the use of micellar catalysis in the context of the catalytic functionalization of carbon–hydrogen bonds. The micelles accumulate catalyst and reactants in their inner volume in such a high local concentration that kinetics are favored. The consequence is that, in most cases, processes that in conventional organic solvents require high temperatures and long reaction times are achieved in milder conditions when micellar catalysis is employed.

## Introduction

The transition-metal-catalyzed C–H activation and functionalization through homogeneous catalysis is one of the most versatile and useful synthetic strategies in modern chemistry.^1^ In many cases, the inertness of such moieties, due to their high values of bond dissociation energies, causes these reactions to be carried out under conditions far from being considered mild or atom-economic. This is the origin of the increasing interest in promoting such processes under more sustainable and environmentally friendly conditions, with optimized values of E-factors.^2^ One major approach is the consideration of alternative, nonconventional solvents as the reaction medium.^3^ This is the case of reactions carried out under solvent-free conditions, or employing ionic liquids, supercritical fluids, fluorous solvents, or water. From the perspective of green chemistry, water as solvent is the most attractive alternative to perform organic reactions,^4^ because it is a nonflammable, nontoxic, and available solvent at nearly no cost. It is also worth mentioning that the environmental factor, or E-factor, introduced by Sheldon,^2^ does not account for water when employed as solvent.

Examples of C(sp^2^ or sp^3^)–H bond activation using water as a reaction medium have been reported.^5^ However, the low solubility of many organic molecules and/or the low stability of many transition metal complexes used as catalysts limit the use of water as a solvent. Very often, most of these reactions display a heterogeneous nature, and are better defined as reactions “on water” better than “in water”.^6^

**Scheme 1:**
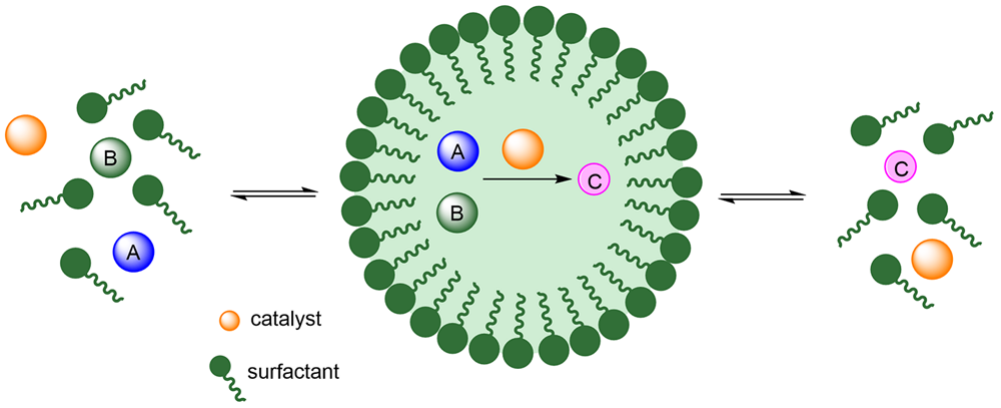
Overview of Micellar Catalysis

In the past two decades, micellar catalysis has emerged as an attractive tool for performing reactions using water as the bulk solvent but providing hydrophobic environments for the reactants and the catalyst.^7^Scheme 1 shows a general view of this strategy. The addition of a surfactant, a molecule containing a polar end and a nonpolar chain, to water originates the spontaneous aggregation into micelles, which can accommodate reactants and catalysts in the inner region, triggering the catalytic reaction given their high local concentration, usually much higher than under homogeneous conditions. The micelles undergo continuous aggregation/disaggregation processes, favoring reactant/product exchange.

In this contribution we aim at providing the current state of the art of the use of micellar catalysis for transition-metal-catalyzed carbon–hydrogen bond functionalization reactions and subsequent C−C bond formation. After a brief introduction about surfactants, a comprehensive description of the different reactions classified according to the type of C–H bond involved is presented. Most if not all examples display better yields and/or milder conditions than the corresponding experiments in organic solvents, as a general feature. We refer to the main corresponding authors in all cases, albeit it is obvious that recognition should be given to all coworkers in each work.

## Micellar Catalysis, Micelles, and Surfactants

Micelles are supramolecular aggregates that are formed by surfactants in water or other water-like media. Surfactants are amphiphilic molecules containing a hydrophobic tail and a hydrophilic head, which allow surfactants to interact with both polar and nonpolar compounds. When surfactants are dissolved in water above a certain minimum concentration, named the critical micellar concentration (CMC), the micelles are formed, showing a hydrophobic core. When employed in catalysis,^7^ the low or nonpolar reactants and catalysts will be concentrated in that inner region of the micelle. The outer hydrophilic surface (cationic, anionic, or neutral) is responsible of the solubility in the polar media. The high local concentration of reactants and catalysts inside the micelle favors their interactions and can increase the reaction rates up to orders of magnitude, compared with corresponding experiments in organic solvents. Furthermore, the rapid equilibrium between the surfactant monomers and the aggregates facilitates the trapping of reactants and release of products in the catalytic reactions (Scheme 1). All these properties of micellar catalysis allow numerous organic reactions to be carried out under mild conditions in water.

**Scheme 2:**
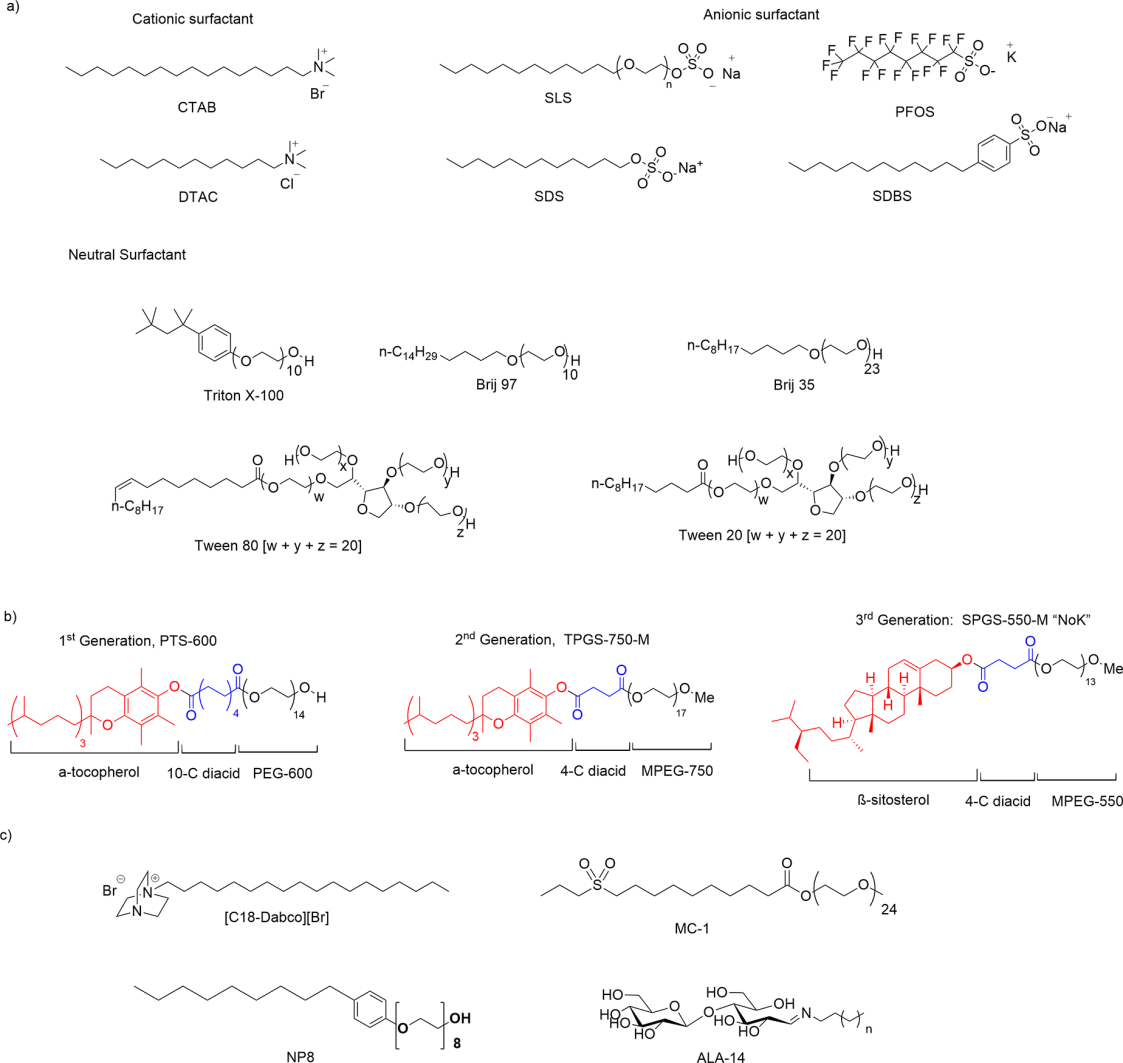
Representative Surfactants: (a) Traditional Cationic, Anionic, and Neutral Surfactants; (b) First Introduced Designer or Green Surfactants; (c) Representative New Designer Surfactants

The concept of micellar catalysis has been known for nearly a century, but it has not been considered as a possible green alternative to traditional homogeneous catalysis until the past few decades.^7^ A number of studies have allowed the development of this strategy, with particular mention to the group of Lipshutz,^8^ which has delivered many examples to promote different organic transformations. Toward that end, a great variety of commercial surfactants and designer surfactants (Scheme 2) have been employed. The tuning of each system with the appropriate surfactant has allowed these reactions, which required hard reaction conditions in traditional media, to be carried out under mild conditions with the same catalyst as in organic solvents.

The designer or *green* surfactants were developed by Lipshutz and appeared in successive generations (Scheme 2).^9^ The first- and second-generation surfactants, PTS and TPGS-750-M, are formulated as racemic vitamin E derivatives, while the third-generation surfactant, Nok or SPGS-550M, is based on natural phytosterol, β-sitosferol. Many designer surfactants have appeared in the literature, and a very complete review article on this topic has been disclosed by Scarso.^9c^

## 1 C(sp)–H Bond Functionalization

### Sonogashira Coupling Reactions

The Sonogashira coupling consists of the carbon–carbon cross-coupling of terminal alkynes with aryl- or vinyl-halides, using palladium(II) as the catalyst and copper(I) as the cocatalyst. This type of reaction has allowed obtaining a wide variety of organic products with biological, pharmaceutical, and industrial interest. In recent years, Sonogashira couplings have been achieved in the presence of several commercial cationic and anionic surfactants and first, second, and third generation designer surfactants (Scheme 2). Actually, Sonogashira couplings have become the most useful and studied method for the C(sp)–C(sp^2^) bond formation in micellar media.

#### Synthesis of Ynones

**Scheme 3:**
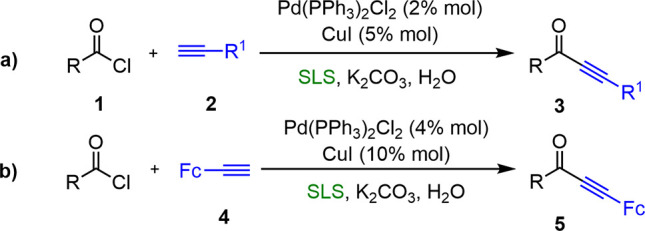
Synthesis of Ynones

First examples of Sonogashira couplings under micellar conditions were tested using sodium lauryl sulfate (SLS) as the surfactant. In 2004, Li and Chen^10^ described a highly effective direct coupling of acid chlorides with terminal alkynes catalyzed by PdCl_2_(PPh_3_)_2_/CuI in the presence of SLS and K_2_CO_3_ as the base. The desired ynones were obtained with yields within the interval 66–99% (Scheme 3a). Later, Lv and coworkers developed a similar method.^11^ A wide variety of ferrocenylethynyl ketones were synthesized by the coupling reaction of ferrocenylethyne with different acyl chlorides using SLS as the surfactant and K_2_CO_3_ as the base (Scheme 3b). In both cases, the presence of a surfactant was essential for the coupling reactions, also assessing its key role in protecting and stabilizing the acyl chloride reactants against hydrolysis.

#### Arylation of Heterocycles

**Scheme 4:**
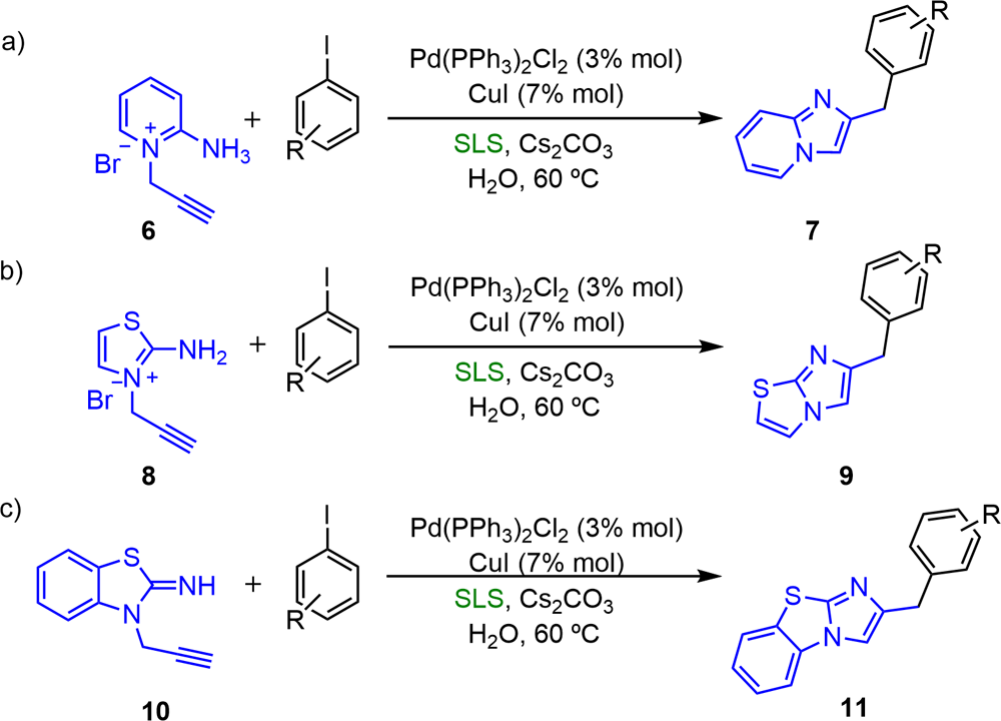
Synthesis of (a) 2-Substituted Imidazo[1,2-*a*]pyridines; (b) 6-Substituted Imidazo[2,1-*b*]thiazoles; (c) 2-Substituted Imidazo[2,1-*b*][1,3]benzothiazoles

Heterocyclic compounds have also been synthesized by the Sonogashira coupling under mild and micellar conditions, following a combination of the cross-coupling process with internal electrophilic cyclization. Bakherad^12^ has succeeded in obtaining several types of heterocyclic compounds, such as imidazopyridines (**7**), imidazothiazoles (**9**), and imidazobenzothiazoles (**11**), by the coupling of aryl iodides with the corresponding aminopyridines, aminothiazoles, and aminobenzothiazole bromides using SLS as the surfactant and Cs_2_CO_3_ as the base at 60 °C (Scheme 4).

In 2021, Taddei reported^13^ the synthesis of 2-substituted indoles by a tandem Sonogashira-cyclization reaction using Pd(OAc)_2_/XPhos as the catalyst, in the absence of copper, employing a 3 wt % TPGS-750-M water solution, affording the desired products with moderate yields.

#### Alkynylation of Arenes

**Scheme 5:**
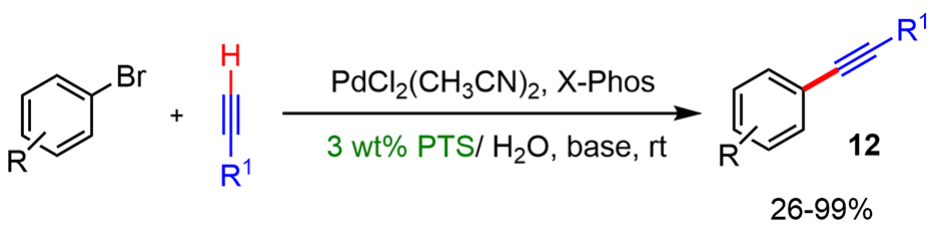
Arene Alkynylation in Water with PTS as Surfactant, with No Copper Added

Coupling reactions of terminal alkynes and aryl bromides or iodides have been carried out in the presence of a wide variety of surfactants, obtaining excellent results. In 2008, Lipshutz reported the first example of a cross-coupling reaction between lipophilic terminal alkynes and aryl bromides using the designer surfactant PTS (Scheme 5).^14^ The addition of this surfactant allowed the efficient formation of desired products in the absence of copper, in water, and at room temperature. For instance, the coupling reaction between phenyl acetate and bromobenzene in the presence of PTS led to 83% isolated products, while without surfactant the conversion was just 34%. Subsequently, Lipshutz improved the efficiency of this type of cross-coupling reaction using the surfactants TPGS-750-M-M14 and Nok.^9^ These results showed the potential of the third generation of surfactants (Nok), replacing the previous ones, which are based on the more expensive vitamin E.

**Scheme 6:**
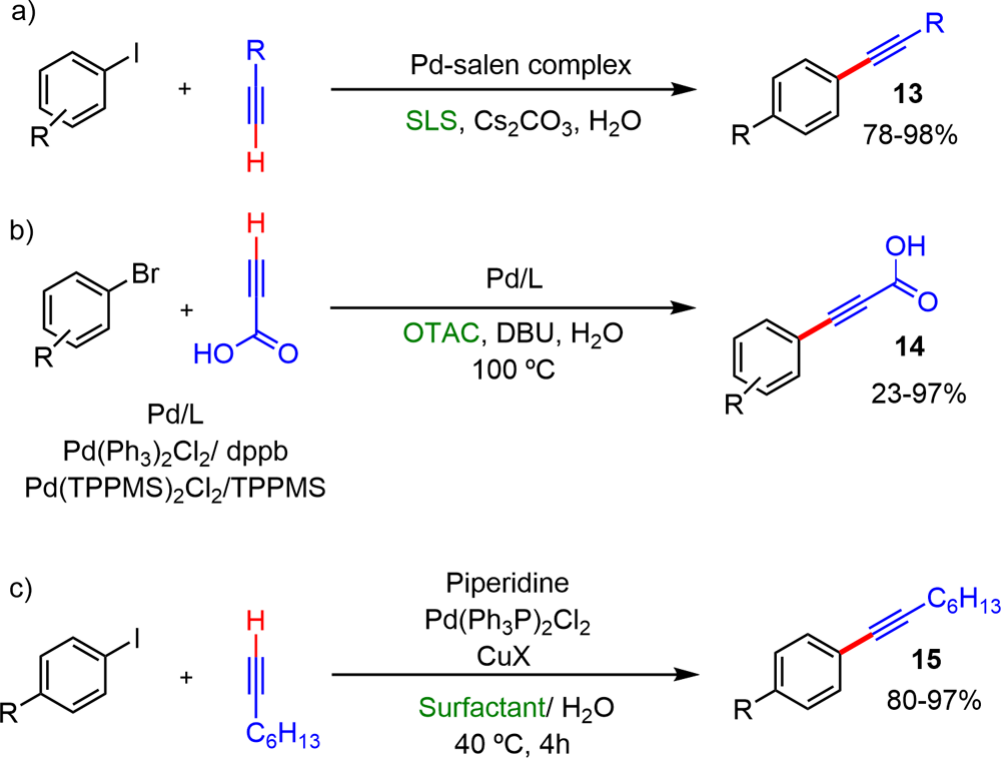
Sonogashira Coupling between Terminal Alkynes and Aryl Halides

The coupling reactions between terminal alkynes and aryl halides have also been described in the presence of different types of common and inexpensive surfactants. For these type reactions, Bakherad^15^ and Lee^16^ independently developed catalytic systems that gave excellent yields in the absence of copper and using SLS (Scheme 6a) and octadecyl trimethylammonium chloride (OTAC) as surfactants (Scheme 6b), respectively. On the other hand, Woo^17^ described the Sonogashira coupling between aryl bromides or iodides and 1-octyne catalyzed by Pd(PPh_3_)_2_Cl_2_ in the presence of commercial surfactants SDS, CTAB, Triton X-100, and Na-Cholate (Scheme 6c). In these cases, the use of Cu(I) salts as a cocatalyst was necessary to achieve high yields. Aryl iodides and 1-octyne led to yields of 80–97%, when SDS or CTAB were used as the surfactant and CuI or CuBr as the cocatalyst (40–60% in the absence of these salts). This group also showed that the nature and the concentration of surfactants has significant influence in the reaction outcome: neutral Triton X-100 was not as efficient as the ionic SDS or CTAB.

New catalytic systems operating in the absence of copper and under micellar conditions have been recently disclosed. The key to the success of such systems stands on a substantial lipophilicity of the catalyst, since it has been demonstrated that it enhances the interaction with the inner hydrophobic core of micelles with subsequent improvement of the catalytic efficiency.^18^

**Scheme 7:**
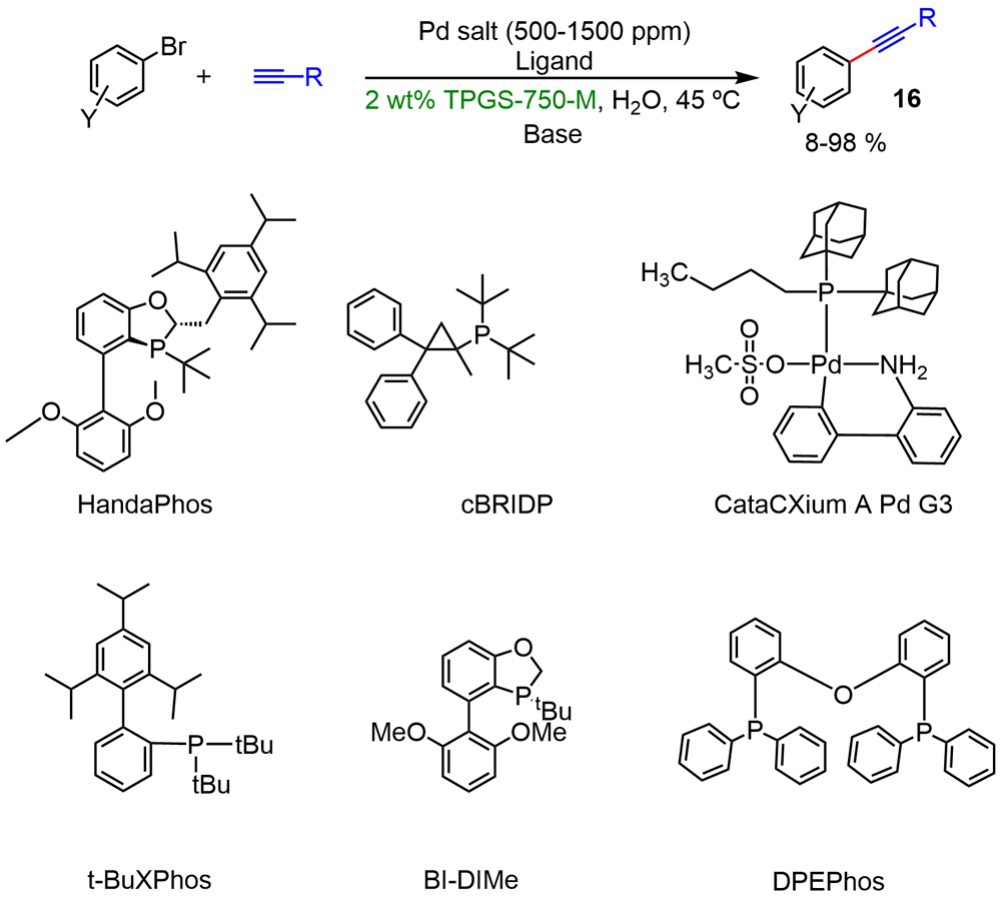
Comparison between HandaPhos and Other Phosphine Ligands

Lipshutz described the new ligand HandaPhos,^19^ a monodentate cyclic phosphine, which led to Sonogashira couplings with catalyst loadings as low as 0.1 mol % (copper-free), and with TPGS-750-M as the surfactant. Later, the commercially available cBRIDP ligand yet allowed the use of the palladium catalyst at the ppm level. Likewise, other catalytic systems based on phosphine ligands, such commercially available catalyst CataCXium A Pd G3, also led to 98% with catalyst loading <1% in TPGS-750-M and using glucose as the additive and THF as a cosolvent.^20^ These studies confirmed the important role that the nature of the ligands performed in the reactions carried out under micellar conditions. On the other hand, the use of other phosphine ligands such as tBuXPhos, BI-DIME, or DPEPhos led to very low yields (Scheme 7).

**Scheme 8:**
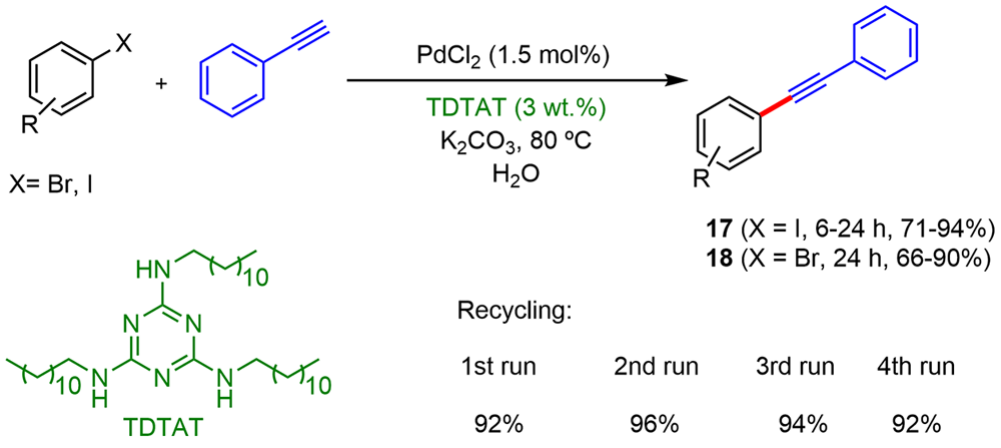
Sonogashira Couplings Using TDTAT Ligand for Several Runs

In recent years, palladium nanoparticles have been described as efficient catalysts for Sonogashira couplings under micellar conditions. For example, Panahi described^21^ the pseudosurfactant TDTAT, containing 2,4,6-trichloro-1,3,5-triazine (TCT) and dodecylamine, which also stabilized Pd NPs as a catalyst for Sonogashira coupling in water. TEM analysis showed that Pd(II) precursors in the presence of TDTAT in water at 80 °C converted into Pd(0) nanoparticles with an average size of ∼3 nm. Also, emulsion droplets containing Pd NPs operated as effective reactors for the C–C coupling reactions, with higher reaction rates. The recycling of these catalysts was verified for five consecutive runs (Scheme 8).

**Scheme 9:**
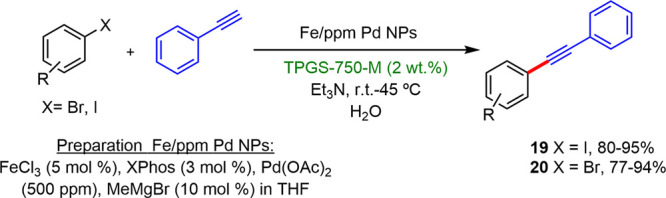
Examples of Products of the Sonogashira Coupling Catalyzed by Fe/ppm Pd NPs

Lipshutz reported a micellar catalytic system based on nanoparticles derived from FeCl_3_ stabilized with the ligand XPhos and with a Pd loading of 500 ppm coupled to nanomicelles of the designer surfactant TPGS-750-M.^22^ This catalytic system induced Sonogashira coupling within the rt to 45 °C interval with excellent yields between 79 and 95% (Scheme 9). The success of this system was associated with the “nano-to-nano” effect, which refers to the natural tendency of MPEG units, present on surface of the spheres of TPGS-750-M, to function as a stabilizing ligand of metallic nanoparticles that are also present as a catalyst in aqueous solutions.^23^ This “nano-to-nano” effect was confirmed by cryo-TEM analyses, which showed a cluster of nanomicelles around metallic NPs.

**Scheme 10:**
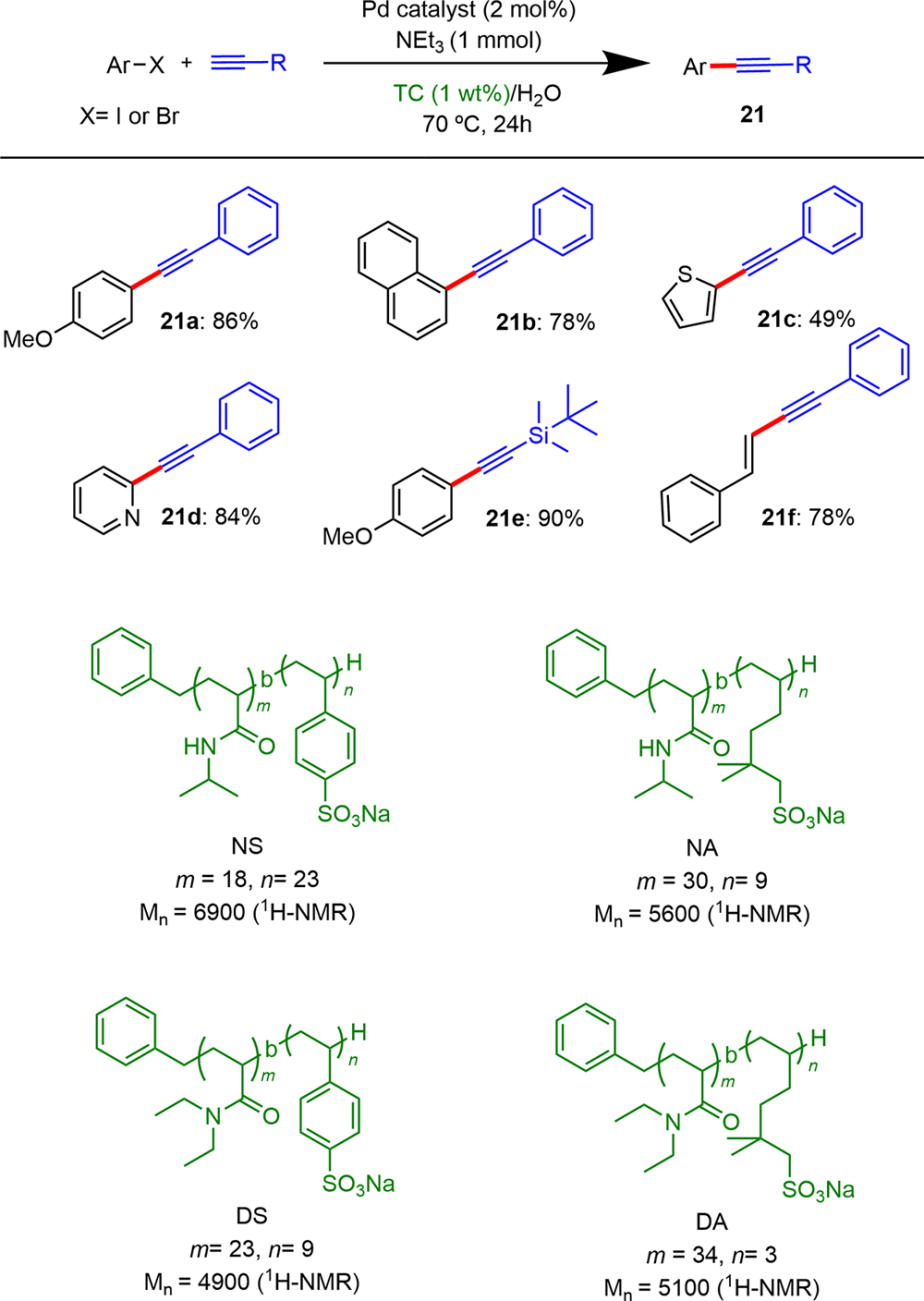
Sonogashira Couplings in Water Using Thermoresponsive Copolymers (TC) as Micelle Precursors

In 2021, Suzuki reported a palladium-catalyzed system using different types of thermoresponsive diblock copolymers as surfactants.^24^ These copolymers formed micelles at temperatures above 50 °C, which were employed as reaction medium for several Pd-phosphine complexes, at 70 °C, leading to excellent yields in the Sonogashira coupling (Scheme 10).

#### Synthesis of Ynamides

Ynamides are terminal active alkynes that have been employed as building blocks for the formation of nitrogen-containing products. The synthesis of ynamines and ynamides has typically involved dry organic solvents because of their moisture-sensitivity and their insolubility in water. However, in the past years, different processes were described employing water as a solvent in the presence of micelles.

**Scheme 11:**
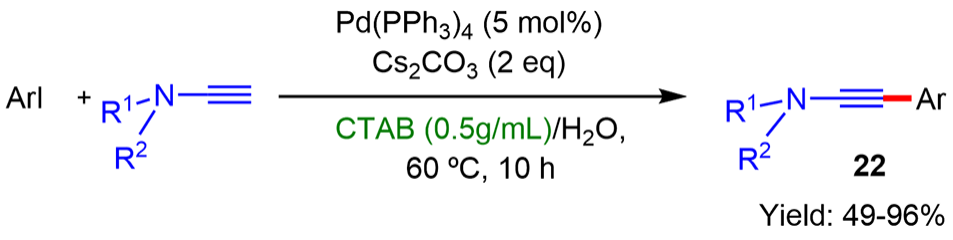
Synthesis of Ynamines by Sonogashira Coupling under Micellar Conditions

In 2021, Zhao reported^25^ the synthesis of ynamides and arylynamines by Sonogashira coupling between ynamines and aryl iodides in water using Pd(PPh_3_)_4_ as the catalyst, Cs_2_CO_3_ as the base, and CTAB as the surfactant. The authors found that this system was tolerant to a broad range of aryl iodides and ynamines featuring both electron-donating and electron-withdrawing groups, affording a wide variety of ynamines **22** in good to excellent yields (Scheme 11). These results suggested that in micellar media, ynamides could be protected from hydrolysis by location at the hydrophobic core of micelles, thus retarding decomposition in water.

### A^3^ Coupling Reactions

The aldehyde-alkyne-amine (A^3^) coupling reaction constitutes a useful synthetic strategy for alkyne functionalization via activation of C–H bonds toward the synthesis of propargylamines. Several contributions have appeared in the past decade for the development of this reaction under micellar conditions.

**Scheme 12:**
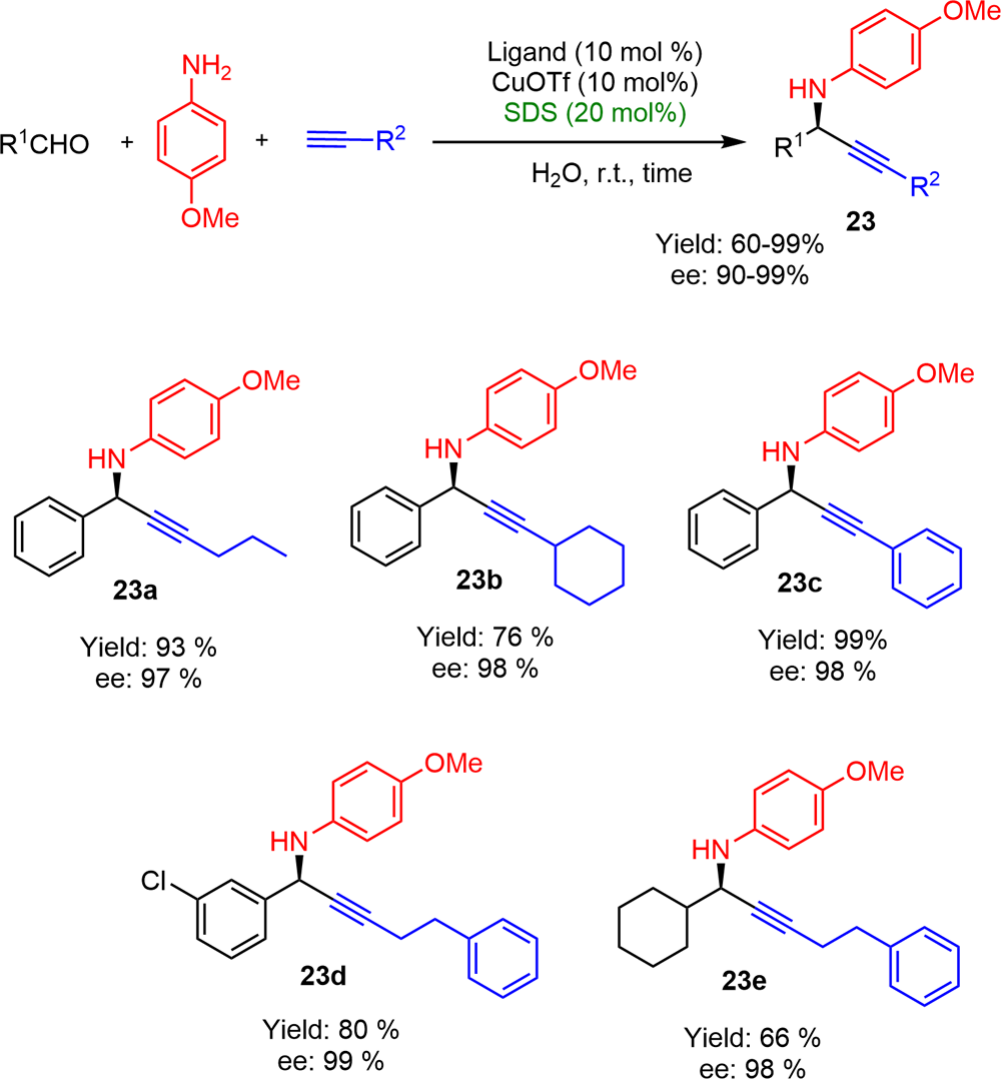
Enantioselective Three-Component Reaction

In 2014, an enantioselective three component reaction of aldehydes, amines, and alkynes in water using bis(imidazoline)Cu^I^ species as catalysts and SDS as the surfactant was reported by Nakamura (Scheme 12).^26^ The reaction took place, in the presence of SDS, with excellent yields, whereas the use of other anionic, cationic, or neutral surfactants such as SLS, CTAB, or Triton X-100 did not provide good results (Table 1). A broad range of aldehydes and alkynes was tested to give optically active propargylamines with excellent yields (60–99%) and enantiomeric excess (90–99%).

**Table 1:**
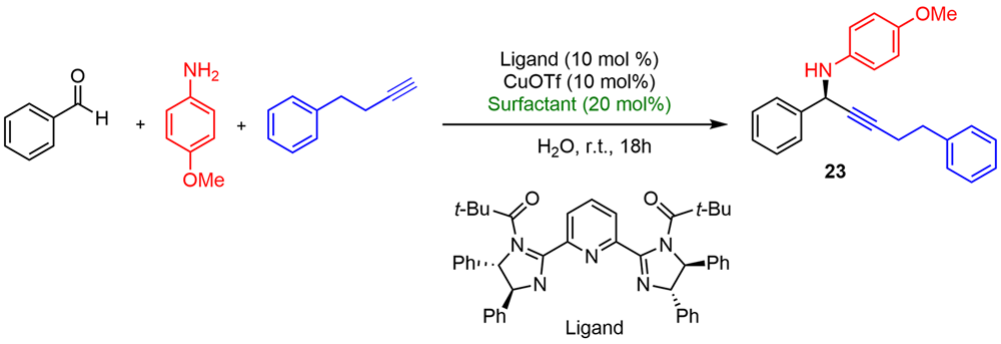
Enantioselective Three-Component Reactions Using Various Surfactant Types

entry	surfactant	yield (%)	ee (%)
1	SDS	99	98
2	CTAB	0	–
3	Triton X-100	17	98
4	SLS	0	–

**Scheme 13:**
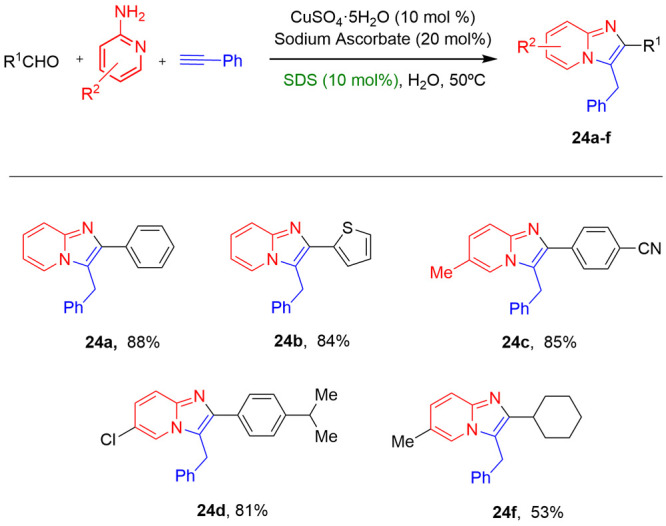
Representative Examples of Imidazo[1,2-*a*]pyridine Obtained by A^3^ Reaction in Micelles

Banerjee later disclosed an efficient synthesis of imidazo[1,2-*a*]pyridine derivatives by A^3^ coupling reaction catalyzed by Cu(II)-ascorbate in aqueous micellar media (Scheme 13) in the presence of SDS.^27^ This system showed a high tolerance for both electron-withdrawing and electron-donating substituents on 2-aminopyridine and benzaldehyde substrates. A wide variety of imidazo[1,2-*a*]pyridine with good yields between 50 and 89% were obtained. The reaction was very slow in the absence of surfactant, yielding only 14% of **24a** at 80 °C after 24 h. This suggested that the micellar “nanoreactors” were necessary to bring together water-insoluble components in their hydrophobic core, thus favoring the reaction to proceed.

## 2 C(sp^2^)–H Bond Functionalization

### Arylation Reactions

One of most useful synthetic strategies to achieve the functionalization of aromatic C(sp^2^)–H bonds is the arylation reactions employing aryl halides and (hetero)arenes. The relative inertness of such bonds requires the use of hard conditions, including high temperatures and/or strong acidic media, with subsequent drawbacks such as tolerance to functional groups. The use of directing groups has been used as an alternative strategy, albeit it does not always solve the problems. Recent studies have shown that micellar catalysis may provide high yields and selectivities under mild conditions.

**Scheme 14:**
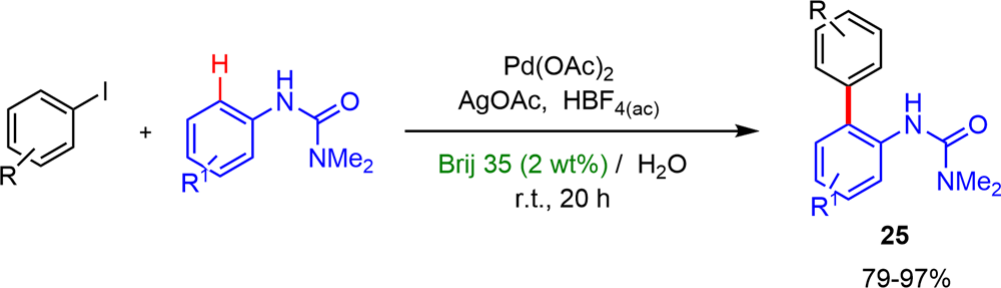
C–H Arylation Process in Micellar Media

**Scheme 15:**
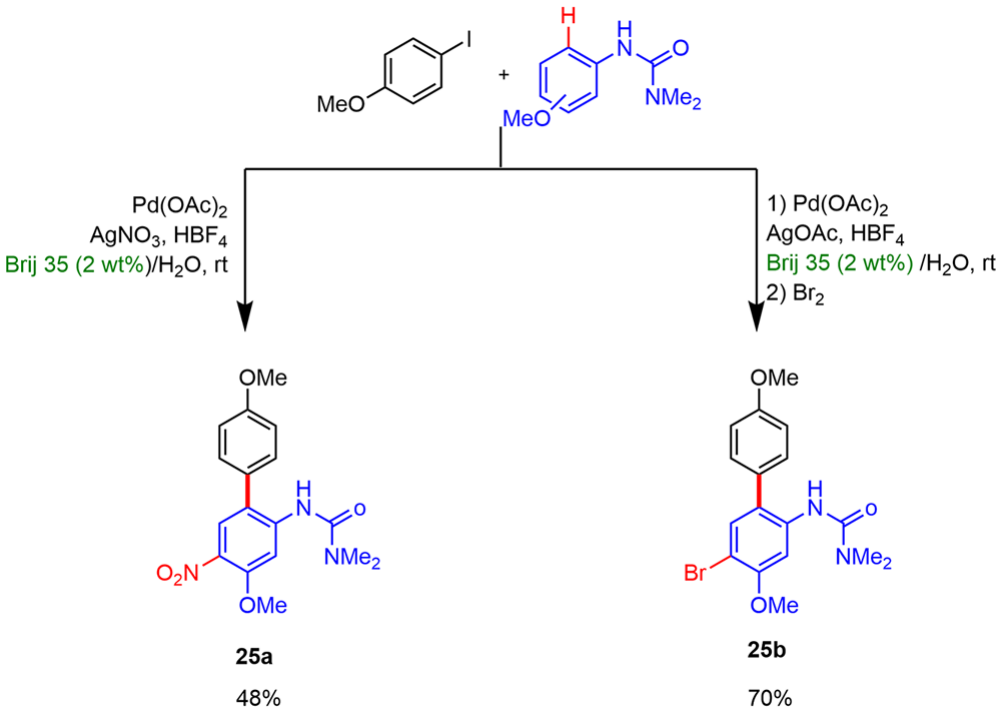
Tandem C–H Arylation/Electrophilic Trapping

In 2010, Lipshutz reported^28^ the first mono C(sp^2^)–H arylation reaction under mild conditions in water. Arylation reactions of aniline derivatives with aryl iodides catalyzed by Pd(OAc)_2_ was performed in the presence of AgOAc and HBF_4_ in 2 wt % surfactant/water solutions at room temperature. Several common and designer surfactants were tested. The best results were reached using Brij 35, which led to yields between 79 and 97% under optimal reaction conditions (Scheme 14). Also, they found that the micellar conditions improved selectivities, since aniline derivatives lacking ortho- or meta-substitutions, which have previously shown to be prone to double arylation, formed products exclusively from monoarylation reaction under these conditions. The limitation of these reactions in water was the use of sterically hindered substrates or electron-deficient ureas. Likewise, a series of tandem processes, like C–H activation/electrophilic trapping, were described under these mild conditions: bromide and nitrate biaryl products were isolated with good yields (Scheme 15).

**Scheme 16:**
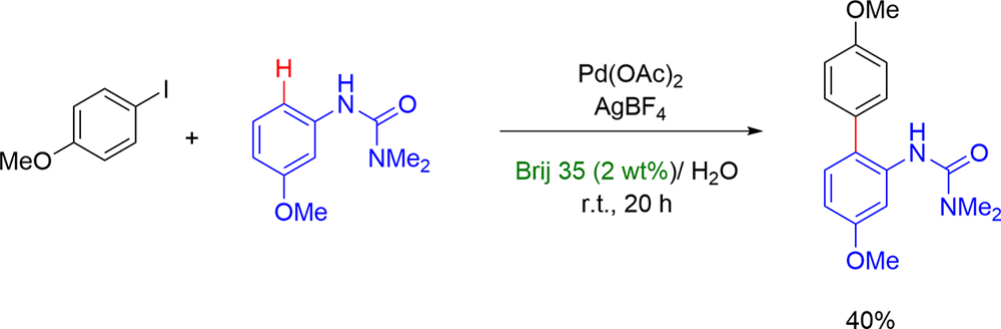
Acid-Free C–H Bond Arylation in Micellar Media

The mechanistic studies showed that the presence of active cationic palladium species was essential for the arylation reaction to proceed at room temperature. When the catalytic system, Pd(OAc)_2_-AgOAc-HBF_4_, was replaced by a commercially available palladium complex, [Pd(MeCN)_4_](BF_4_)_2_, the reaction was inhibited. However, when the catalytic system was substituted by the system Pd(OAc)_2_-AgBF_4_, biarylated products were obtained with moderate yields around 40% without the assistance of any acid or coordinated ligand (Scheme 16).

**Scheme 17:**
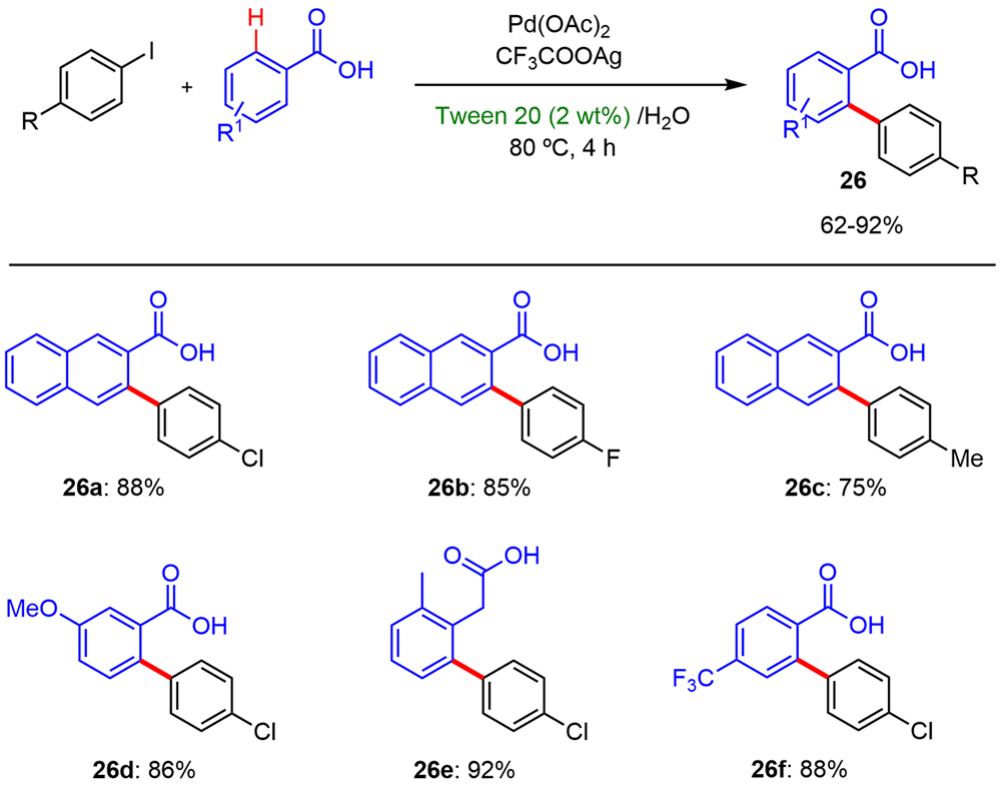
Arylation Reaction of Carboxylic Acid Derivatives under Micellar Conditions

Ren reported^29^ an efficient Pd-catalyzed carboxylate-directed C–H arylation reaction of aryl carboxylic acids with iodobenzenes in 2 wt % surfactant/water solutions where different commercial surfactants were used. The presence of these surfactants improved the solubility of the starting materials in reaction media and allowed carrying out this type of reaction at a lower temperature (80 °C) than those previously reported (>100 °C), using conventional organic solvents. The desired products were isolated with yields between 62 and 92% (Scheme 17).

The effect of the nature of surfactants in the reaction outcome is illustrated in Table 2. For example, the neutral surfactants such as Brij 35, Tween 80, or Tween 20 were effective for the C–H arylation of aryl carboxylic acids, whereas ionic surfactants such as SDS or CTAB led to low conversions. The best results were achieved using Tween 20 as the micelle precursor.

**Table 2:**
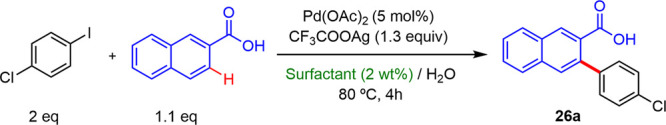
Scope of Surfactants for Pd-Catalyzed Carboxylate-Directed C–H Arylation in Water

entry	surfactant	conversion (%)
1	none	0
2	Tween 80	82
3	Tween 20	87
4	Tween 40	77
5	Tween 60	55
6	Brij 35	67
7	SDS	10
8	HTAB	40

**Scheme 18:**
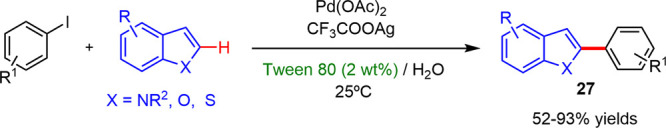
C2-Arylation of Indoles, Benzofuranes, and Benzothiophenes at Room Temperature in the Presence of Tween 80

The C–H activation/arylation reactions of indoles, benzofuranes, and benzothiophenes is also of interest, albeit most of the reported methods have generally showed regioselectivity issues unless directing groups, high temperatures (100–150 °C), or acid cosolvents are employed. In 2015, Ren reported^30^ the mild, efficient, and C2 selective palladium-catalyzed arylation reaction of indoles, benzofurans, and benzothiophenes with iodobenzenes at room temperature in the presence of Tween 80 as the surfactant. The products were synthesized with good yields, and in a selective manner toward that position, with no detection of the C3 arylation derivatives (Scheme 18).

**Scheme 19:**
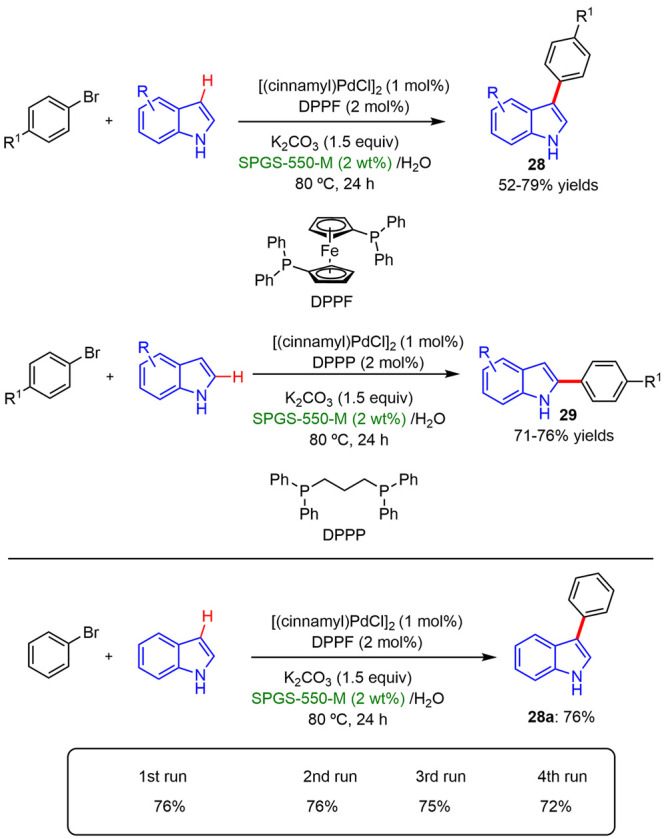
Selective C3/C2-Arylation of (NH)-Indoles in the Presence of SPGS-550-M (Top); Reusability of the Surfactant Solutions (Bottom)

Subsequently, Kumar described^31^ a selective C3/C2 arylation of indoles using the designer surfactant SPGS-550-M (Nok) in the presence of [cinnamyl)]PdCl_2_]/phosphine ligand, as the catalyst in water at 80 °C. The micellar medium allowed this process to be carried out under mild conditions with high yields, and outstanding regio- (C3 vs C2) and chemoselectivity (C vs N) control. A high functional group tolerance was also observed. The nature of the phosphine ligand displays the key role for achieving site-selectivity. DPPF and DPPP ligands were the most effective in promoting the arylation at C3–H and C2–H, respectively. Also, the surfactant solutions could be recycled and reused without compromising product yields (Scheme 19).

**Scheme 20:**
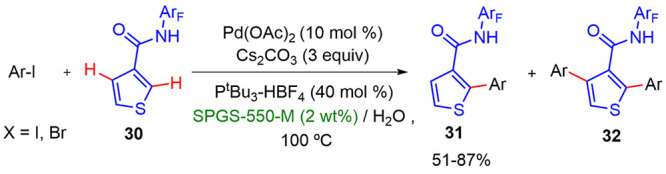
Arylation of Thiophenes in Micellar Medium

**Scheme 21:**
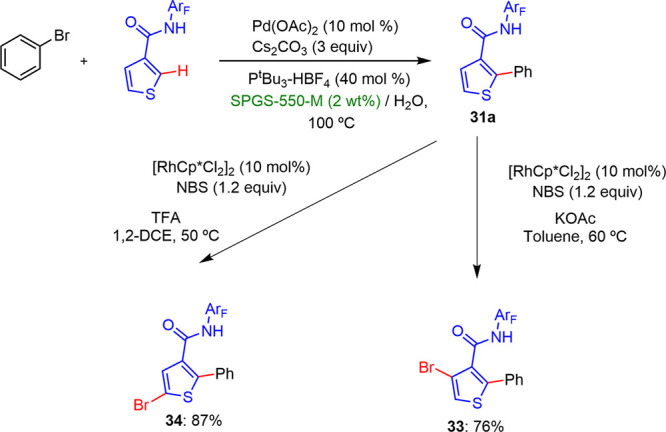
Sequential Regioselective Functionalization of Thiophenes

Tan described a Pd-catalyzed arylation of 3-substituted thiophenes in a regioselective manner when performed in water/surfactant media (Scheme 20).^32^ These conditions provided the desired C2-arylated thiophene derivatives with high yields and selectivities. The phosphine ligand, P^*t*^Bu_3_, performed a key role in selectivity control toward monoarylation products, limiting the functionalization to the 4-position. In this work, it was found that the starting material and desired product showed a greater stability in water than in the previously used organic solvents. The addition of different types of surfactants improved the efficacy and led to better yields. The best results were reached with SPGS-550-M (Nok). Whereas electron-withdrawing substituted aryl halides led to excellent yields and selectivities, those bearing electron-donating groups showed lower oxidative addition reaction rates, increasing the ratio of the bisarylated products. Moreover, the functionalization of positions C4 and C5 was achieved with high regioselectivities. The formation of 2,3,4-substituted thiophenes was only found when the arylation reaction was tested using a rhodium catalyst and KOAc as a weak base under basic conditions. On the other hand, in the presence of a strong acid such as TFA, only the functionalization of position 5 was observed. The 2,4,5-substituted thiophenes were obtained with the best yields under these conditions (Scheme 21).

**Scheme 22:**
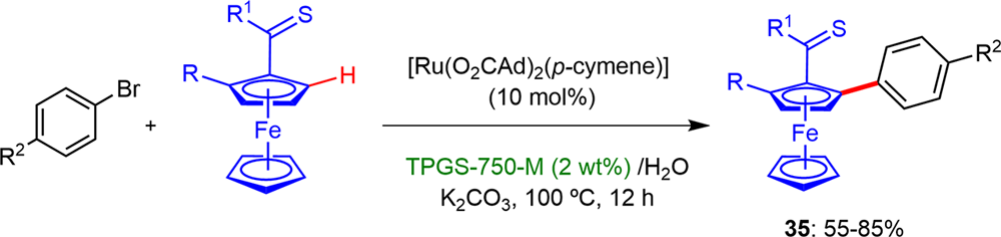
Arylation of Ferrocenes under Micellar Conditions

In 2019, Ackermann described^33^ the first example of chemoselective arylation of ferrocenes catalyzed by a ruthenium complex in water through micellar catalysis. The presence of a single component ruthenium catalyst and K_2_CO_3_ as the base gave the optimal results, in a process assisted by weakly coordinating thiocarbonyls in an aqueous solution of surfactant, TPGS-750-M (Scheme 22). This reaction showed full tolerance with valuable functional groups such as chloro, bromo, ester, ketone, or aldehyde, with a wide variety of mono- and bis-substituted ferrocenes. Also, the designer surfactant could be recycled up to four times without affecting the reaction yields.

### Alkenylation Reactions

Another useful synthetic method for the formation of C–C bonds from C(sp^2^)–H aryl bonds is the alkenylation of arenes. Examples reported in the literature in conventional media usually need an elevated temperature and anhydrous acidic conditions, as well as high pressures of CO or O_2_ as the oxidant. Also, these studies showed the pernicious effect of water as an inhibitor reagent. Only very recently it has been demonstrated that micellar catalysis can be employed toward the alkenylation reaction of C(sp^2^)–H bonds in water.

**Scheme 23:**
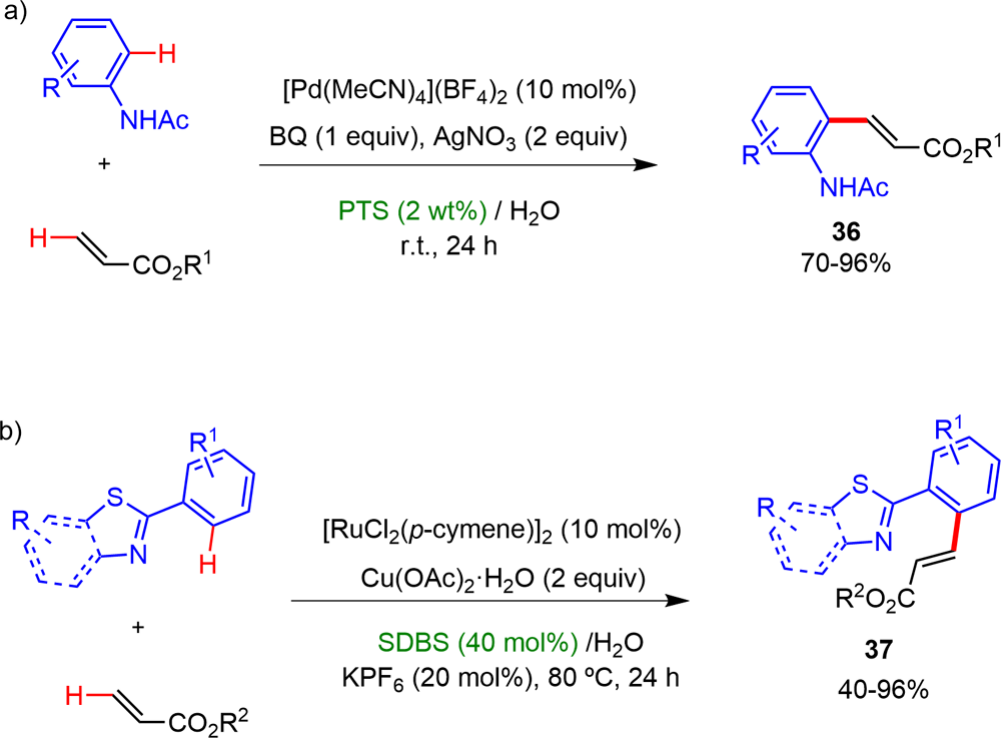
Alkenylation Reactions in Micellar Media

In 2010, Lipshutz and Nishikata reported^34^ the first example of an efficient catalytic Fujiwara–Moritani alkenylation reaction of C–H bonds between anilide and acrylate ester. The complex [Pd(MeCN)_4_](BF_4_)_2_ was employed as the catalyst and AgNO_3_ and 1,4-benzoquinone as additives without an external acid at room temperature in surfactant/water solutions as reaction media. The surfactant PTS led to the best results, with high yields and regioselectivities (Scheme 23a). Later, Ding developed^35^ an efficient ruthenium(II) catalyzed regioselective *ortho-*oxidative C–H bond alkenylation of substituted 2-arylbenzo[*d*]thiazoles and 2-aryltihiazoles with diverse acrylates using Cu(OAc)_2_ as the oxidant in water in the presence of SDBS at 80 °C (Scheme 23b). In the case of 2-arylbenzo[*d*]thiazoles, the alkenylation reaction afforded the desired products with excellent yields and regioselectivities in the presence of both electron-rich as well as electron-deficient derivatives. However, the alkenylation of 2-arylthiazoles gave alkylated products with high yields but moderate selectivities.

### Mizoroki–Heck Reactions

**Scheme 24:**
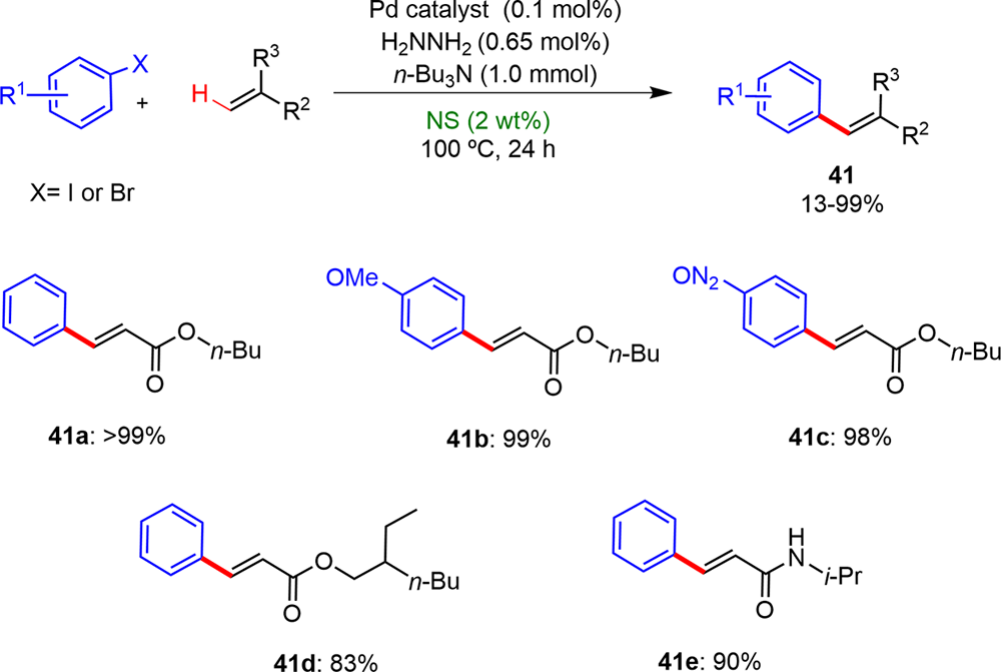
Mizoroki–Heck Reactions in Micellar Media with Thermoresponsive Copolymers as Micelle Precursor See Scheme 10 for NS.

In 2019, Suzuki and Tsai described Pd-catalyzed Mizoroki–Heck reactions between aryl iodides and terminal alkenes in water at 70 °C using thermoresponsive block copolymers **39** and **40** as surfactants (Table 3).^36^ A broad variety of aryl iodides and alkenes with electron-donating and electron-withdrawing substituents were tested, affording a wide range of products in good and excellent yields. They found that like conventional surfactants, the nature of these copolymers also affected to efficiency of the reactions. The reactions carried out using the copolymer **39**, which presents an anionic tail, were more efficient than those with **40**, lacking such tail, as the surfactant (Table 3). The use of thermoresponsive block copolymers shown in Scheme 10 was also described.^24^ A number of Pd complexes were examined, with the Mizoroki–Heck reaction giving the corresponding products in excellent yields (Scheme 24) with low palladium loadings (0.1 mol %).

**Table 3:**
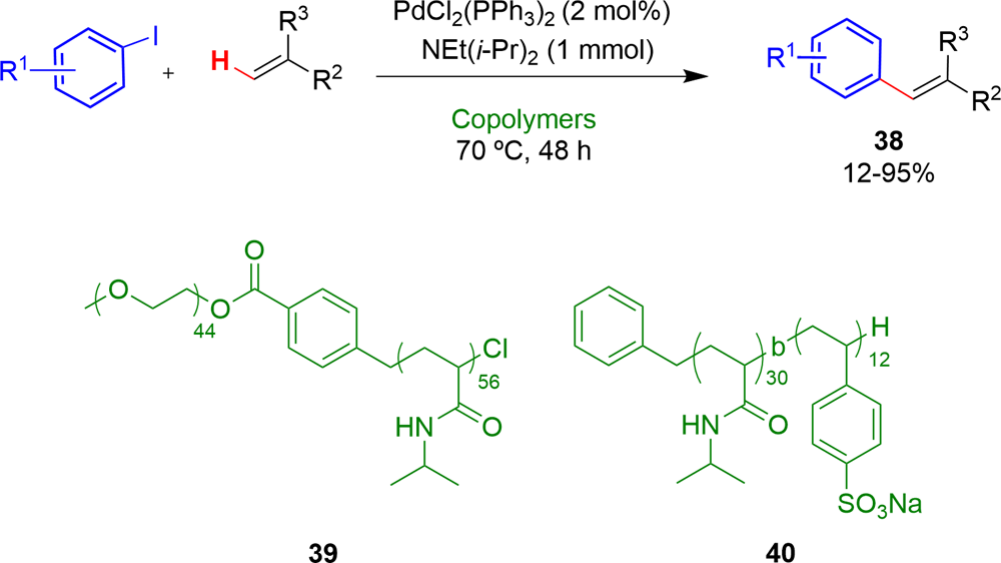

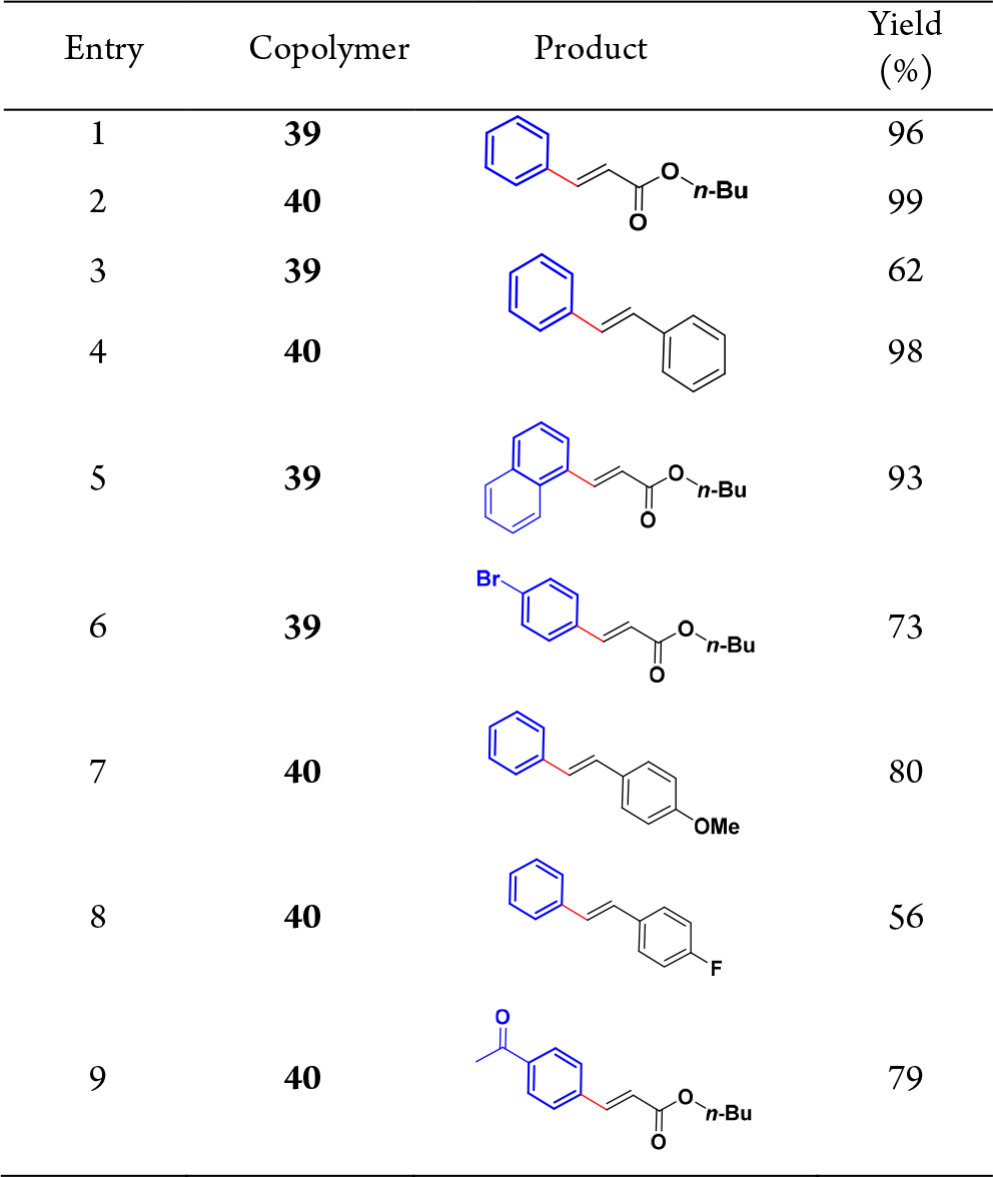
Representative Examples of Mizoroki–Heck Reactions with Various Aryl Halides and Alkenes in Water

**Scheme 25:**
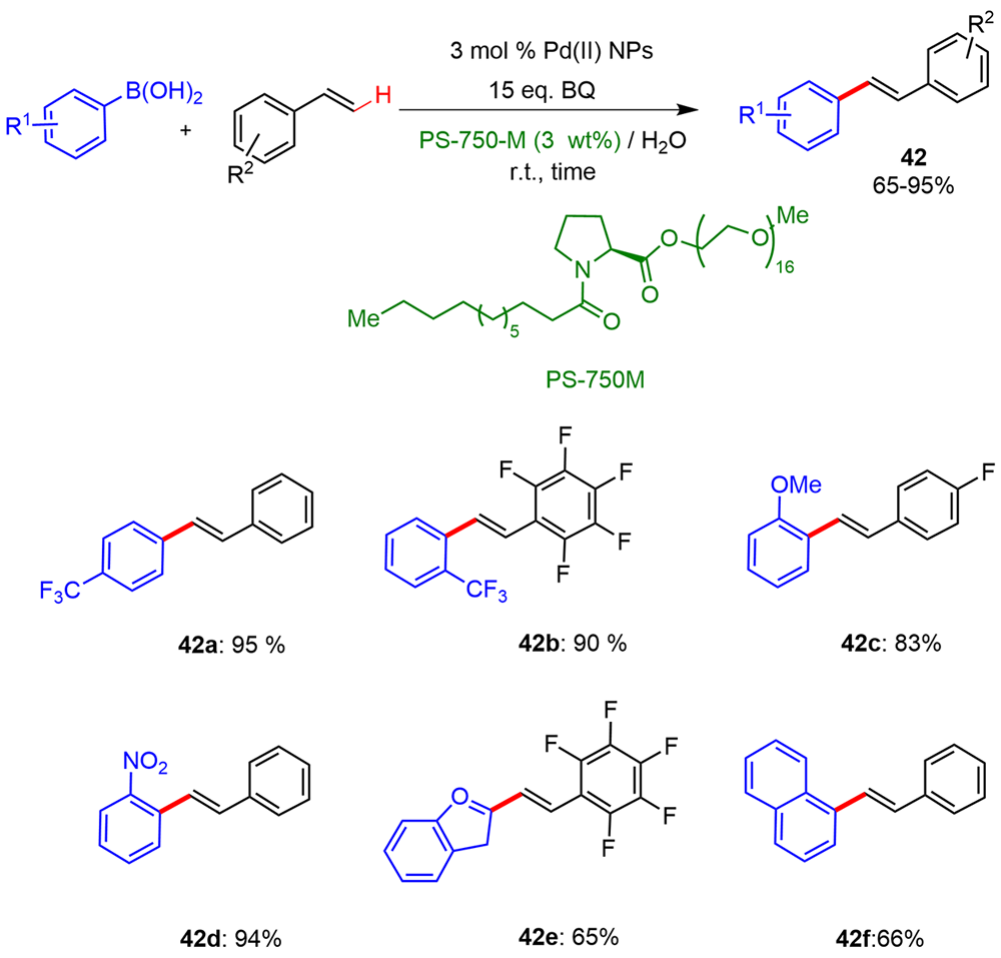
Coupling Reactions between Styrenes and Aryl-Boronic Acids under Micellar Conditions

Handa reported the development of stable, phosphine ligand-free, and catalytically active Pd(II) nanoparticles generated from Pd(OAc)_2_ and coupled to micelles with last generation surfactant PS-750M. The Pd NPs were efficient catalysts for the coupling reactions of styrene and aryl-boronic acids in an aqueous solution of PS-750 M at room temperature, which are unusual conditions for these coupling reaction types (Scheme 25).^37^

**Scheme 26:**
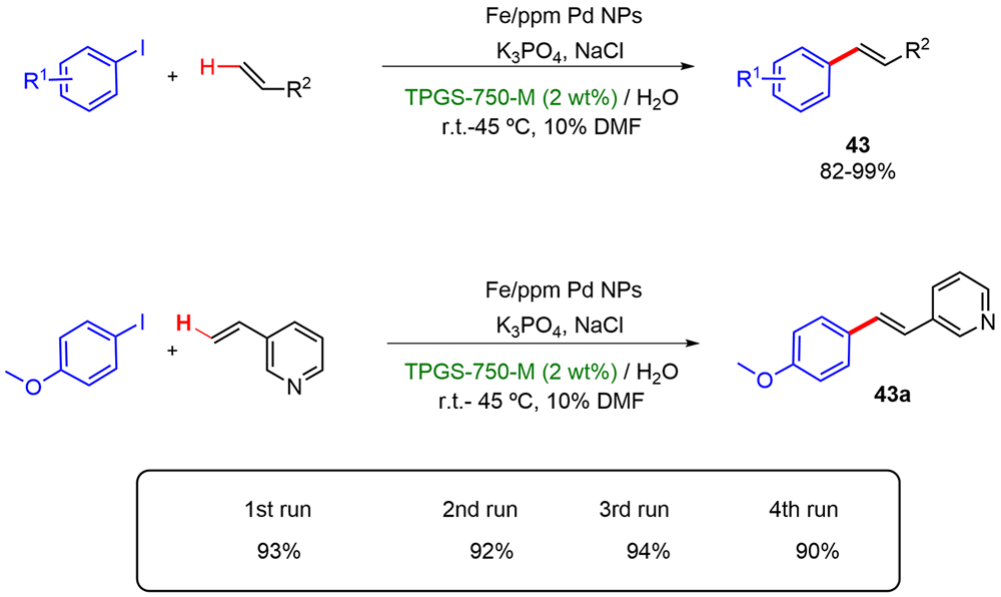
Arylation Reaction of Alkenes Catalyzed by Fe/ppm Pd NPs

Subsequently, Lipshutz described a new catalytic system, formed by Fe nanoparticles derived from FeCl_3_ containing ppm levels of Pd ligated by P^*t*^Bu_3_, capable of efficiently performing the arylation reaction of alkenes in 2 wt % TPGS-750-M/water solution using DMF as a cosolvent between room temperature and 45 °C. Excellent yields (82–99%) of a variety of products with different functional groups were thus obtained (Scheme 26).^38^ This group found that micellar conditions were necessary to efficiently carry out these catalytic reactions since the aqueous conditions altered the morphology of NPs, transforming inactive spherical NPs to rod-shaped, catalytically active NPs. Moreover, the catalytic system could be recycled, leading to similar yields after four consecutive cycles.

### Heteroarene Alkylations

**Scheme 27:**
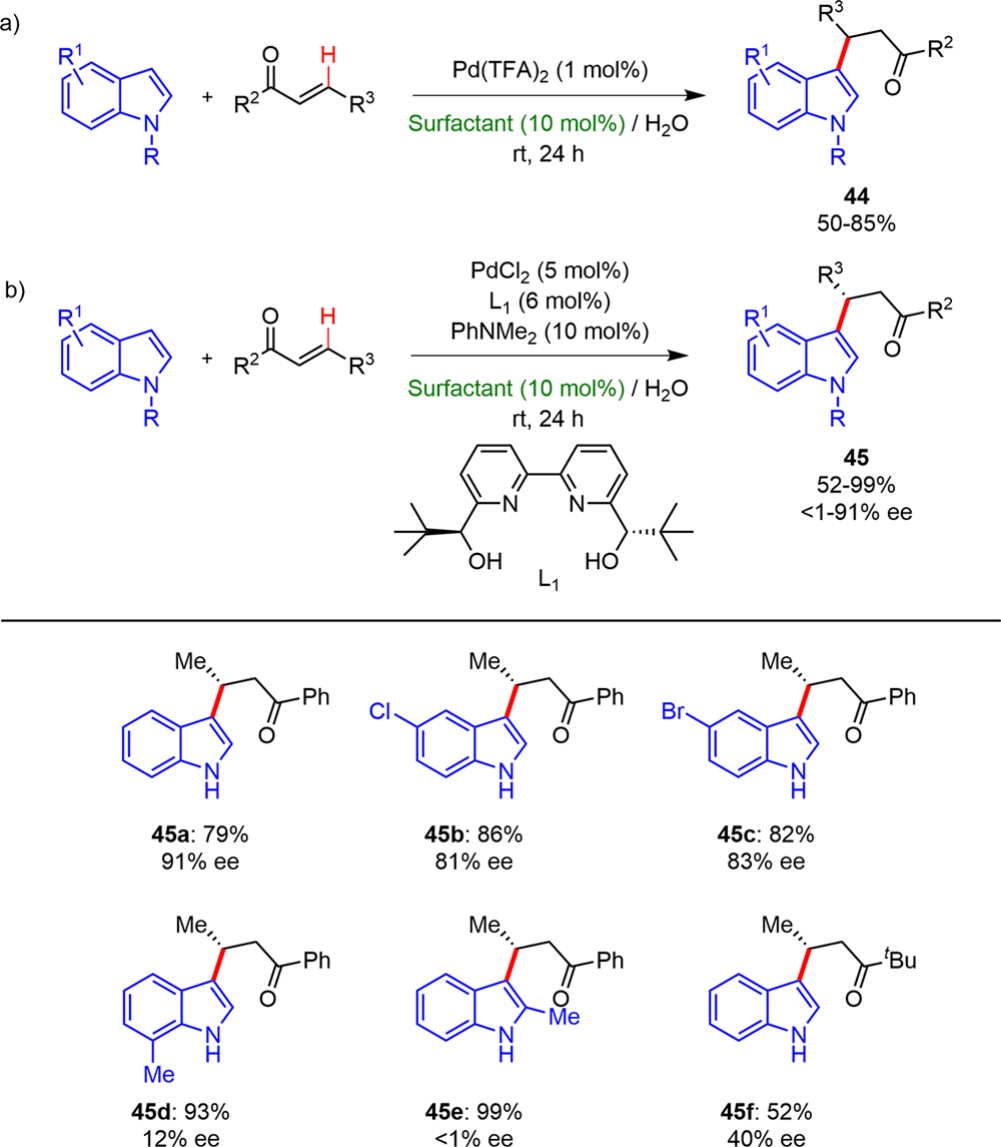
Alkylation of Indoles under Micellar Conditions

The transition-metal-catalyzed alkylation reactions of indoles with alkenes constitutes a well-established synthetic tool for C(sp^2^)–C(sp^3^) bond formation. In 2015, Kobayashi reported a selective C3-alkylation of 1*H*-indoles with α,β-unsaturated compounds catalyzed by an electrophilic palladium(II) complex in a micellar media at room temperature (Scheme 27a).^39^ The alkylated products were obtained with yields between 50% and 85% and excellent regioselectivities under optimal reaction conditions. Moreover, they found that the presence of an anionic surfactant, such as SDS or SBDS, was essential to induce the alkylation reaction in good yields, and nonionic or cationic surfactants did not facilitate the transformation. The authors suggested that the cationic Pd(II) species was stabilized inside of micelles generated by anionic surfactants, therefore inhibiting the Pd(II) degradation to inactive catalytic Pd(0).

The same group later described the enantioselective version, employing palladium/bipyridine (**L1**) as a chiral ligand, SDS as the surfactant, and PhNMe_2_ as the additive at room temperature (Scheme 27b).^40^ Phosphine-based sterically hindered bidentate ligands were found useless for this reaction in the micellar medium, SDS/H_2_O. However, when chiral 2,2′-bipyridines (**L1**) were used along with different palladium(II) salts such as PdCl_2_, Pd(OAc)_2_, PdBr_2_ and PdCl_2_(MeCN)_2_, the desired products were achieved with yields between 52% and 99%, whereas enantiomeric excesses were dispersed (1–91%).

**Scheme 28:**
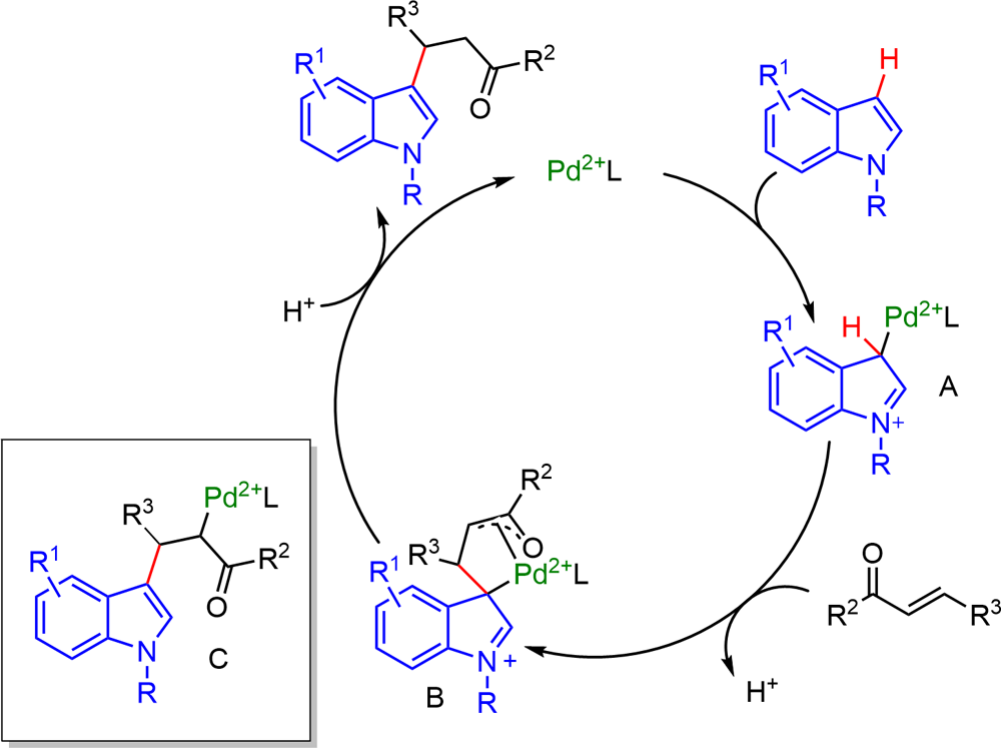
Proposed Catalytic Cycle of Electrophilic C–H Functionalization with Cationic Pd(II)

Kobayashi proposed a mechanism for this transformation in micellar media (Scheme 28),^40^ involving electrophilic palladation with an electron-deficient palladium(II) species to generate an indolyl-palladium intermediate (**A**), which reacted with α,β-unsaturated compounds to form C-bonded palladium(II) enolates (**B** or **C**). Despite the intrinsic propensity of **C** to undergo β-hydrogen elimination, they proposed **B** to be stabilized in the micellar surface.

### Arene Acylations

**Scheme 29:**
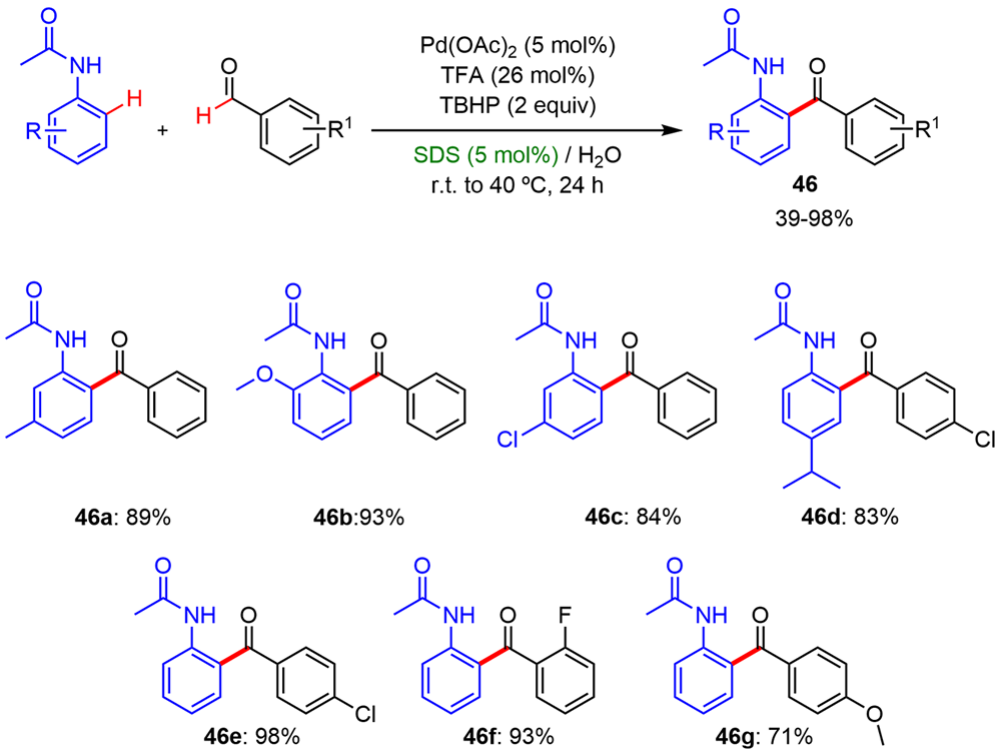
*ortho*-C–H Acylation Reaction under Micellar Conditions

In 2013, Novák developed a new and efficient palladium-catalyzed C–H bond activation method for the synthesis of aryl ketones from anilides and aldehydes in a micellar medium.^41^ This oxidative coupling was performed in an aqueous solution of SDS using TBHP as the oxidant and TFA as the additive at temperatures between 25 and 40 °C. The system worked for an array of combinations (Scheme 29), including substituted anilides as well as aldehydes bearing electron-donating or electron-withdrawing substituents, leading to the corresponding aryl ketones with good and excellent yields. Also, the authors found that the micellar conditions were essential for the success. The use of conventional solvents such as CH_3_Cl, CH_2_Cl_2_, and toluene gave conversions in the range of 22–30%, at variance with those observed with the SDS–water system (63–93%).

**Scheme 30:**
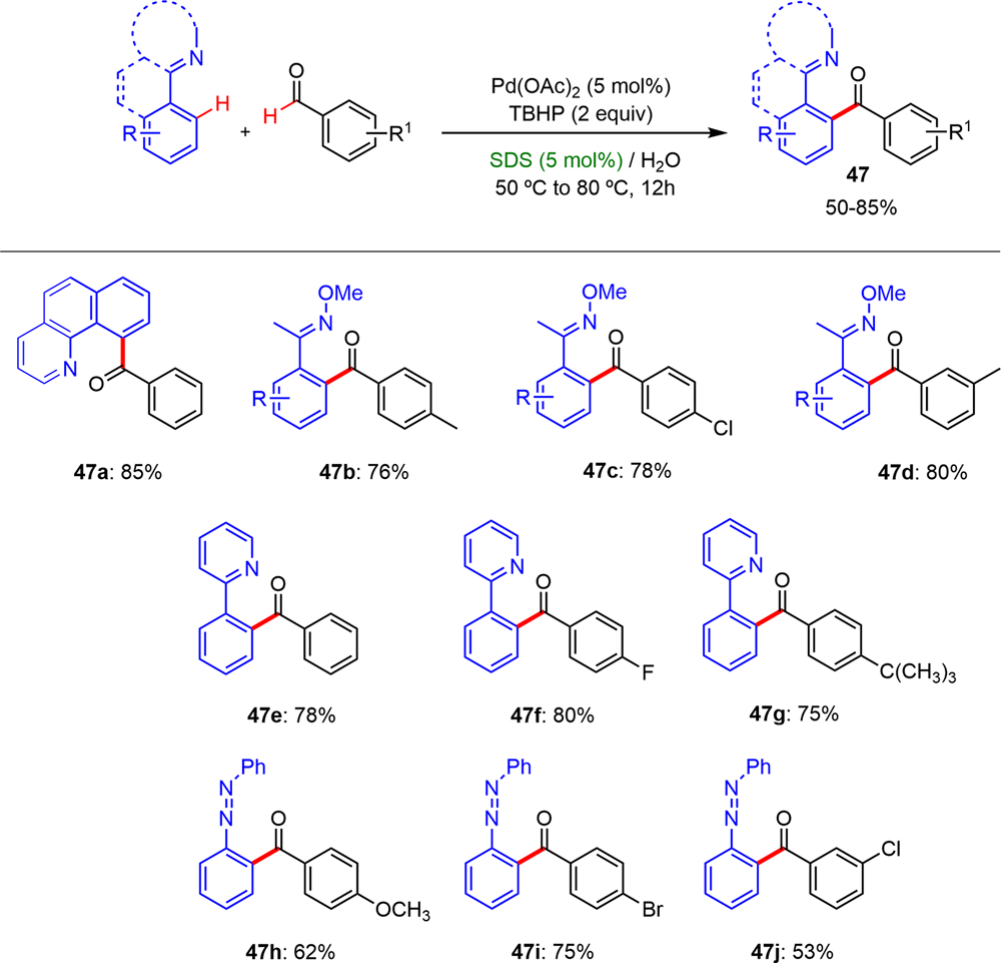
Expansion of the Acylation Reaction under Micellar Conditions

Later, Deng and Xiao succeeded in expanding the field of direct C–H acylation with aromatics aldehydes in a micellar medium (Scheme 30).^42^ They described the synthesis of diaryl ketones by a palladium-catalyzed *ortho*-C–H acylation reaction between aromatic aldehydes and aromatic oximes, azo aromatics compounds, and arylpyridines using TBHP as the oxidant in an aqueous solution of SDS at temperatures between 50 and 80 °C. The diaryl ketones were isolated with good yields between 50% and 85% under optimal reaction conditions (Scheme 30). Furthermore, they found that these acylation reactions exhibited excellent regioselectivity and a wide tolerance toward both electron-rich as well as electron-deficient functional groups.

## 3 C(sp^3^)–H Bond Functionalization

The direct formation of C–C bonds via C(sp^3^)–H activation bonds remains one of the most challenging research topics nowadays. The low reactivity and high thermodynamic stability of C(sp^3^)–H bonds are the main drawbacks to circumvent. In past decade, several research groups have developed different catalytic systems capable of carrying out the direct formation of C–C bonds from C(sp^3^)–H moieties under micellar conditions.

### Alkane Functionalization by Carbene Insertion

**Scheme 31:**
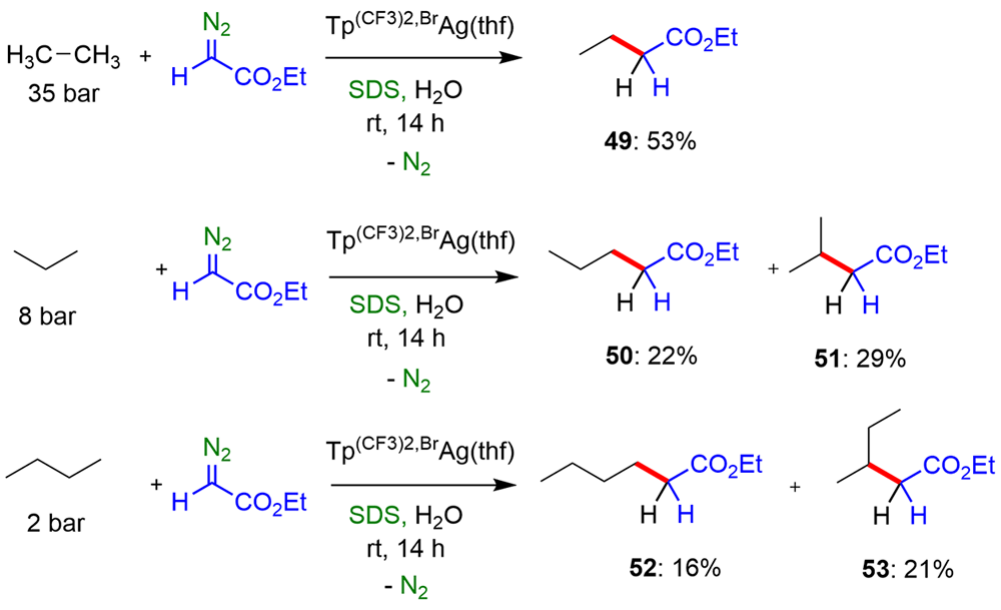
C2–C4 Gaseous Alkane Functionalization in Water at Room Temperature

In 2019, our group reported the first example of functionalization of methane in water at room temperature employing micellar catalysis.^43^ We employed a silver-based catalyst, Tp^(CF3)2,Br^Ag(thf), capable of performing the formation of ethyl propionate from methane (160 bar) and ethyl diazoacetate (EDA) at room temperature in the presence of the surfactants SDS and PFOS. Ethyl propionate was afforded in 10% and 14% of yields, respectively (Table 4). When SDS was used as the surfactant, part of the ethyl diazoacetate initially added led to functionalization of the hydrocarbon chains of the surfactant as well as to the formation of ethyl glycolate (HOCH_2_CO_2_Et) from H_2_O. On the other hand, the use of fluorinated surfactant avoided the functionalization of the fluorinated chains. Other gaseous alkanes such as ethane, propane, and butane were also functionalized following the similar methodology, reaching EDA-based yields in the range of 37–53% (Scheme 31).

**Table 4:**
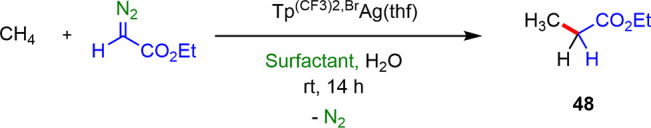
Methane Functionalization in Water–Surfactant Mixtures

entry	surfactant	yield (%)
1	none	0
2	Triton X-100	0
3	DTAC	0
4	TPGS-750-M M	2
5	SDS	10
6	PFOS	14

### Monoarylation Reactions of C(sp^3^)–H Bonds

**Scheme 32:**
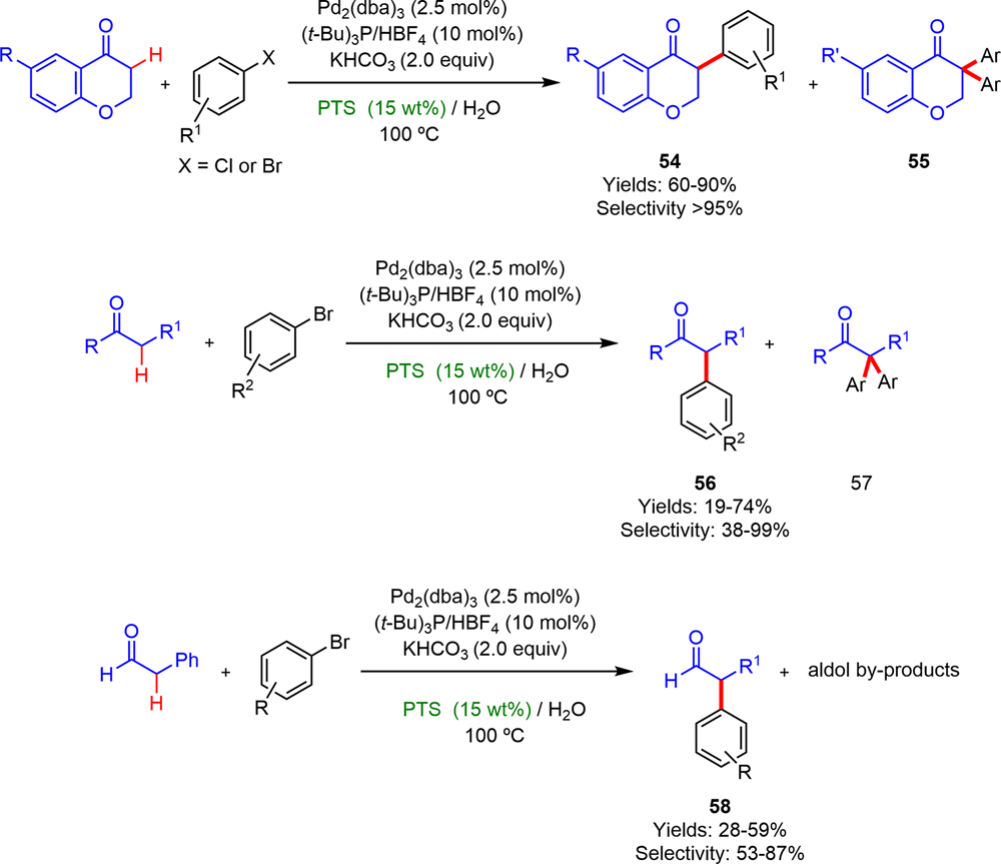
Pd-Catalyzed α-Arylation of Carbonyl Derivatives with Aryl Halides in PTS/H_2_O

In 2011, Rossi described the highly selective direct C–H α-monoarylation reaction between 4-chromanones, ketones, or aldehydes with aryl halides in the presence of Pd_2_(dba)_3_/P^*t*^Bu_3_/HBF_4_ as the catalyst and KHCO_3_ as the base in 15 wt % surfactant/water solution at 100 °C (Scheme 32).^44a^ The best results were reached using PTS, and the optimal conditions led to the desired isoflavanones with yields within 60–90% values. A decrease in yields and selectivities was observed when the α-monoarylation reactions were performed with aldehydes or ketones as substrates. However, these results surpassed those previously reported in nonmicellar media, using dioxane/H_2_O as reaction medium.^44b^

**Figure 1:**
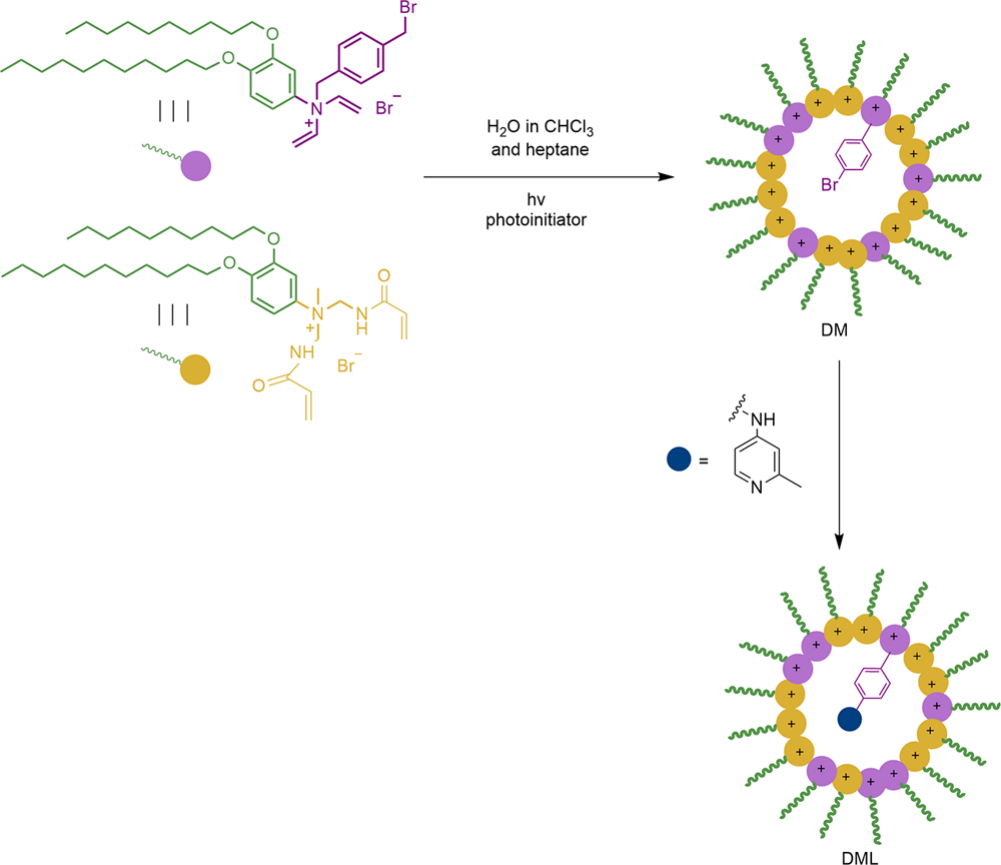
General synthesis of cross-linked micelle-supported ligand.

**Scheme 33:**
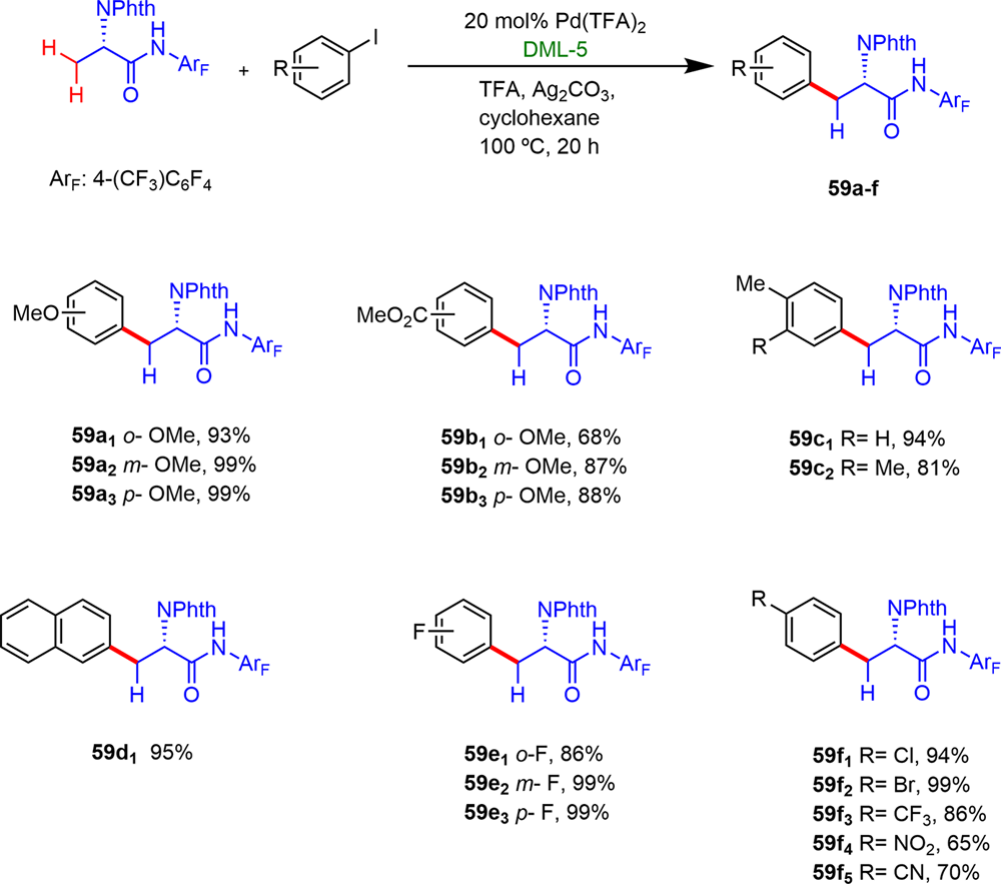
Pd-Catalyzed C(sp^3^)–H Arylation Using DML-5

Jones later reported the first example of C(sp^3^)–H monoarylation catalyzed by a cross-linked reverse micelle supported palladium(II) catalyst.^45^ Reverse micelles were designed to promote selectivity trends influenced by the steric and electronic effects inside the micelle core (Figure 1). They showed that in this new catalytic system both micelles and supported palladium catalyst exhibited a high tolerance and compatibility with electron-donating and electron-withdrawing substituents of the aryl iodides located in *ortho-*, *meta-*, and *para*-positions (Scheme 33), leading to a series of products with high yields (70–99%) and selectivities (74–99%). DML-5 led to the best results, where DML stands for double-tail micelle with ligands, and 5 being the number of the core size.

**Scheme 34:**
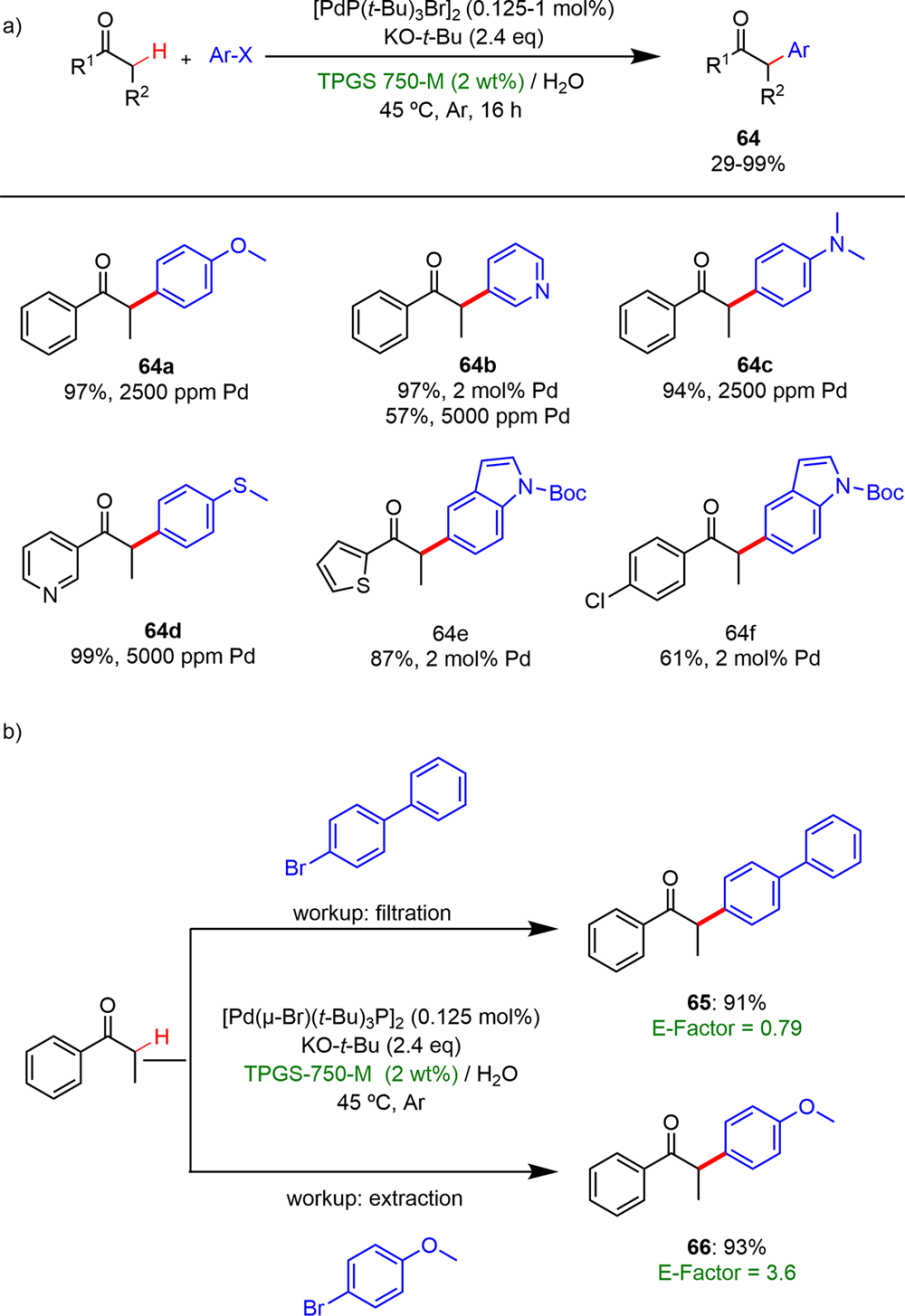
(a) C(sp^3^)–H α-Arylations under Aqueous Micellar Conditions; (b) E-Factors Calculated for Selected Examples Catalyst loadings giving in ppm or percentage.

In 2021, Lipshutz and co-workers described a new methodology for the α-arylation of aryl and heteroaryl ketones in an aqueous solution of the surfactant TPGS-750-M using a highly active and commercially available precatalyst, [Pd(μ-Br)P(*t*-Bu)_3_]_2_.^46^ The reactions were performed with a broad range of aryl halides and ketones (Scheme 34a). Altering the aryl halides showed that electron-rich and neutral substrates afforded arylated products with yields 73–99% using Pd loading lower than 0.5 mol %. However, electron-poor halides were not that effective, and they required higher catalyst loading (2 mol %). When the arylation reactions were carried out using aryl methyl ketones and dialkyl ketones as starting materials, good yields were also reached, although furan or thiophene led to double α-arylation products for most aryl halides. The E-factors calculated for two examples are shown in Scheme 34b, showing the high degree of *greenness* that these transformations display in comparison with the counterparts carried out in conventional reaction media.

### The Beneficial Effect of Micellar Catalysis in Residual Metal

One important feature of the use of micellar catalysis is the observance of a significant decrease of the residual metal in the products. This is crucial in terms of the usage of the products for pharma industry. For example, the system shown in Scheme 34b led to a residual metal level of 6.6 ppm of Pd.^46^ This value is below the FDA maximum daily dose value of 10 ppm of such metal. The same behavior has been reported for copper-free Sonogashira couplings (Scheme 7), where the amounts of palladium were an order of magnitude below those reported for experiments carried out with 1–5% catalyst loading.^19a,19b^ A last, representative example corresponds to Mizoroki–Heck coupling catalyzed by metal nanoparticles, which led to the lack of detection of metal (below the detection limit) in the products,^38^ making the route suitable for the synthesis of a precursor of galipinine.

## Conclusions

In the context of the search for sustainable and environmentally benign strategies within the area of transition-metal-catalyzed C–H bond functionalization, micellar catalysis has emerged as an alternative to conventional methods. The addition of surfactants to water generates micelles that accommodate catalysts and reactants in the inner region in such a way that the high local concentration usually triggers the kinetics of the reaction, therefore decreasing temperature and reaction times when compared with the corresponding reactions in conventional (usually organic) reaction media. Alkyl, aryl, alkenyl, or alkynyl C–H bonds have been modified upon translating the known reactions in organic solvent to micellar media, in most cases with a significant shift toward greener conditions. The advances achieved in the past two decades point toward the significant value of this strategy in the incoming years.
